# Assessment of Covalently Binding Warhead Compounds in the Validation of the Cytomegalovirus Nuclear Egress Complex as an Antiviral Target

**DOI:** 10.3390/cells12081162

**Published:** 2023-04-14

**Authors:** Julia Tillmanns, Sigrun Häge, Eva Maria Borst, Julia Wardin, Jan Eickhoff, Bert Klebl, Sabrina Wagner, Christina Wangen, Friedrich Hahn, Eileen Socher, Manfred Marschall

**Affiliations:** 1 Institute for Clinical and Molecular Virology, Friedrich-Alexander University of Erlangen-Nürnberg (FAU), 91054 Erlangen, Germany; 2 Institute of Virology, Hannover Medical School, 30625 Hannover, Germany; 3 Lead Discovery Center GmbH (LDC), 44227 Dortmund, Germany; 4 The Norwegian College of Fishery Science UiT, The Arctic University of Norway, 9037 Tromsø, Norway; 5 Institute of Anatomy, Functional and Clinical Anatomy, FAU, 91054 Erlangen, Germany

**Keywords:** human cytomegalovirus, regulation of viral replication, nuclear egress complex (NEC), conditional expression of viral core NEC proteins, antiviral targeting based on NEC 3D structures, covalently binding warhead compounds, pronounced NEC-directed antiviral activity

## Abstract

Herpesviral nuclear egress is a regulated process of viral capsid nucleocytoplasmic release. Due to the large capsid size, a regular transport via the nuclear pores is unfeasible, so that a multistage-regulated export pathway through the nuclear lamina and both leaflets of the nuclear membrane has evolved. This process involves regulatory proteins, which support the local distortion of the nuclear envelope. For human cytomegalovirus (HCMV), the nuclear egress complex (NEC) is determined by the pUL50–pUL53 core that initiates multicomponent assembly with NEC-associated proteins and capsids. The transmembrane NEC protein pUL50 serves as a multi-interacting determinant that recruits regulatory proteins by direct and indirect contacts. The nucleoplasmic core NEC component pUL53 is strictly associated with pUL50 in a structurally defined hook-into-groove complex and is considered as the potential capsid-binding factor. Recently, we validated the concept of blocking the pUL50–pUL53 interaction by small molecules as well as cell-penetrating peptides or an overexpression of hook-like constructs, which can lead to a pronounced degree of antiviral activity. In this study, we extended this strategy by utilizing covalently binding warhead compounds, originally designed as binders of distinct cysteine residues in target proteins, such as regulatory kinases. Here, we addressed the possibility that warheads may likewise target viral NEC proteins, building on our previous crystallization-based structural analyses that revealed distinct cysteine residues in positions exposed from the hook-into-groove binding surface. To this end, the antiviral and NEC-binding properties of a selection of 21 warhead compounds were investigated. The combined findings are as follows: (i) warhead compounds exhibited a pronounced anti-HCMV potential in cell-culture-based infection models; (ii) computational analysis of NEC primary sequences and 3D structures revealed cysteine residues exposed to the hook-into-groove interaction surface; (iii) several of the active hit compounds exhibited NEC-blocking activity, as shown at the single-cell level by confocal imaging; (iv) the clinically approved warhead drug ibrutinib exerted a strong inhibitory impact on the pUL50–pUL53 core NEC interaction, as demonstrated by the NanoBiT assay system; and (v) the generation of recombinant HCMV ∆UL50-ΣUL53, allowing the assessment of viral replication under conditional expression of the viral core NEC proteins, was used for characterizing viral replication and a mechanistic evaluation of ibrutinib antiviral efficacy. Combined, the results point to a rate-limiting importance of the HCMV core NEC for viral replication and to the option of exploiting this determinant by the targeting of covalently NEC-binding warhead compounds.

## 1. Introduction

The human cytomegalovirus (HCMV) belongs to the subfamily of β-herpesviruses and is distributed worldwide with a high seroprevalence of 40% to 95%, depending on socioeconomic status [1]. A characteristic of all herpesviruses is the latency leading to a life-long persistence in the organism interspersed with recurrent symptoms through reactivations [2]. The course of HCMV infection may substantially vary depending on the immune status of the infected person. Immunocompetent individuals usually remain asymptomatic or show mild courses, whereas HCMV in newborns and immunosuppressed patients, such as transplant recipients and cancer and human immunodeficiency virus type 1 (HIV-1)-infected patients, can lead to severe symptoms, organ failure and even life-threatening situations [3,4]. To date, HCMV is far the most frequent vertically transmitted viral infection during pregnancy, which consequently may lead to stillbirth or developmental defects in the newborn and infant, such as microcephaly, deafness or mental retardation [5,6]. Currently available drugs for the treatment of HCMV infections are restricted to the inhibition of distinct viral targets, resulting in the frequent occurrence of drug resistance and limited options of combination therapy [4,7]. In addition, most of the anti-HCMV drugs have side effects and are limited in use, e.g., none of them is approved for application during pregnancy [8] or may resolve latent infection [9].

During lytic replication within the host cells, newly amplified viral genomes are packaged in capsids within the nucleus followed by final maturation of the infectious particles in the cytoplasm. The large diameter of HCMV capsids (approximately 130 nm) prevents their exit via nuclear pore complexes and thus necessitates a capsid transport across the nuclear membrane via the highly regulated and complex multi-stage process of nuclear egress [10,11,12,13]. As a crucial determinant of the herpesviral replication cycle, nuclear egress is conserved between α-, β- and γ-herpesviruses, leading to a massive reorganization of the nuclear envelope [10,11,14,15,16,17]. Key elements of the nuclear egress complex (NEC) are two viral proteins, pUL50 and pUL53, for HCMV, referred to as the core NEC. This serves as a central platform for the formation of a multicomponent NEC, including core NEC-associated regulatory factors and kinases that localize at the inner nuclear membrane (INM). The multicomponent NEC involves emerin, p32/gC1qR, protein kinase C (PKC), cyclin-dependent kinase 1 (CDK1), possibly also CDK2 and further CDKs, the viral kinase vCDK/pUL97 and the peptidylprolyl cis/trans isomerase Pin1 [16,18,19,20,21,22,23,24,25]. In particular, these kinases are of major importance for nuclear egress through their site-specific phosphorylation of lamins. This leads to the disruption of the nuclear lamina in a Pin1-dependent manner, ultimately allowing the capsids to reach the INM [26], and for further steps of maturation through the nuclear envelope and the cytoplasm [16]. The integration of regulatory protein kinases into the nuclear egress process, in particular, vCDK/pUL97, has led to the specific consideration of kinase inhibitors as putative blocking agents of viral nucleocytoplasmic release [24,25,27,28]. Recently, covalently binding kinase inhibitors, referred to as warhead compounds, have attracted the specific interest of researchers and have already entered the various levels of clinical applications [29]. Warheads are mechanistically based on a strongly reactive functional group, such as an α-, β-unsaturated carbonyl group. In most cases, they act through a two-step mechanism [29], in which, first, a high-affinity small molecule ligand, building a drug-like scaffold, reversibly associates with its biological target. Then, the warhead moiety is brought into close proximity to an accessible nucleophilic residue at the biological target, generally a cysteinyl group (-SH), ultimately leading to covalent coupling. Through this spontaneous reaction, the biological target, i.e., the catalytic site of a kinase, according to the original concept, is thereby inactivated. This interaction depends on the specific localization and surrounding of the targeted cysteine, as this influences the accessibility, protonation state and reactivity, which is crucial for an appropriate chemical structure of the warhead [30]. Of note, the property of covalent target binding is mediated through the reactive, unsaturated group (acceptor of Michael addition reaction), whereas target specificity is mainly provided by the nature of the drug scaffold (various chemical classes). Thus, to date, huge efforts have been undertaken to equip warheads with a certain target selectivity, in ideal terms with a monoselectivity, but these endeavors regarding warhead design and target specificity are still in their infancy [29]. Based on the fact that most warheads are directed to structurally exposed cysteine residues of their target proteins, which can be found in a huge number of putative primary and secondary targets, true monoselectivity of warheads is difficult to achieve and currently appears to be rather the exception than the rule ([31]; B.K./Lead Discovery Center, personal communication).

Concerning novel targeting strategies of anti-HCMV drugs, our group and many other researchers have studied a variety of different options of direct-acting and host-directed antivirals [27,32,33]. Very recently, we focused on the NEC as a highly promising target, which is composed of viral core components (core NEC) and, additionally, NEC-associated host and virus proteins (multicomponent NEC) [8,24,34,35,36,37,38,39,40]. A crucial step towards an improved level of discovery of NEC-directed small molecules was our experimental resolution of crystallization-based 3D structures of α-, β- and γ-herpesviral core NECs [41,42,43]. Notably, we identified highly interesting cysteine residues in some of these NEC proteins, which were considered with respect to their accessibility for covalently binding small molecules and thus the possibility of their acting as inhibitors of protein–protein interactions (PPIs).

Generally, it is very attractive to generate NEC-directed PPI inhibitors, since the high-affinity core NEC interaction stands in the center of the nuclear egress process, which is strictly conserved, in terms of NEC regulator functions and structures, among the different herpesviruses. The membrane-anchored groove protein pUL50 of HCMV (and its homologs of other herpesviruses) interacts in a hook-into-groove-like manner with the nucleoplasmic pUL53 (or herpesviral homologs), the latter containing a nuclear localization signal (NLS) [44]. Previous studies of our group demonstrated that pUL50 plays a crucial role in multiple NEC protein interactions and in viral replication efficiency [45]. Building on this, it appeared plausible that the viral core NEC may represent an attractive new antiviral target, and, in fact, specific approaches of target validation have already identified NEC-inhibiting small molecules. These are able to suppress the hook-into-groove interaction of pUL50–pUL53. Moreover, experimental evidence has been provided that such a block in core NEC formation leads to strictly reduced viral replication efficiency [40,46]. On this basis, we hypothesized that the group of covalently binding warheads may represent a promising new type of inhibitors of viral core NEC heterodimerization. As warheads were originally developed as kinase inhibitors [30,47,48,49,50], our focus was directed to the idea that their α-, β-unsaturated carbonyl group may likewise interact with further, so far unconsidered viral target proteins, particularly in those cases in which they carry an exposed cysteinyl group. In regard of the PPIs of the HCMV core NEC, selected warhead compounds were investigated for this inhibitory activity to further specify this novel antiviral targeting concept. Thus, in the present study, we investigated the antiviral potential of a selection of experimental and clinically approved warhead drugs. Antiviral activity was elucidated in terms of a putative inhibitory impact on the viral NEC protein localization, the block of core NEC interaction and the specificity parameters compared to other drugs and other herpesviruses. Moreover, we generated a recombinant HCMV that allowed for the controlled conditional expression of both core NEC components to mechanistically investigate the effects of core NEC-directed antiviral compounds. Combined, several lines of our findings suggest an initial demonstration of the NEC-directed inhibitory potential of selected warhead compounds.

## 2. Materials and Methods

### 2.1. Cell Culture and Virus Infection

Primary human foreskin fibroblasts (HFFs, own repository of primary cell cultures), HFF-UL50 cells [39], MRC-5 embryonic lung fibroblasts and human embryonic kidney epithelial 293T cells (HEK293T; CRL-3216, ATCC, Manassas, VA, USA) were maintained at 37 °C, 5% CO_2_ and 80% humidity. Culture media for HFF, HFF-UL50, MRC-5 (MEM) and 293T cells (DMEM) were supplemented with 10% fetal bovine serum (FBS-12A, Capricorn Scientific, Ebsdorfergrund, Germany), 1 × GlutaMAX^TM^ (35050038, Thermo Fisher Scientific, Waltham, MA, USA) and 10 µg/mL gentamicin (22,185.03, SERVA, Heidelberg, Germany). For the cultivation of HFF-UL50 cells, tetracycline-negative FBS (FBS-TET-12A, Capricorn Scientific, Ebsdorfergrund, Germany) was used and, additionally, 500 µg/mL geneticin was added (G418, 10131035, Thermo Fisher Scientific, Waltham, MA, USA). The expression of pUL50 in the HFF-UL50 cells was induced by addition of 500 ng/mL doxycycline (Dox; D9891, Sigma-Aldrich, St. Louis, MO, USA), which was refreshed at least every 3rd day (d). For HCMV infection of HFF-UL50, the cells were induced with Dox 1 d prior to infection to induce pUL50 expression. HFF or HFF-UL50 cells were infected with stocks of HCMV AD169 [51], AD169-GFP [52], TB40-IE2-YFP [37,53] or AD169-derived recombinant virus ΔUL50-ΣUL53. After incubation for 90 min at 37 °C, the inoculum virus was replaced by fresh medium. For transfection of 293T cells, polyethylenimine (PEI) was used as described before [54] using PBS instead of HBS.

### 2.2. Genetic Recombination and Reconstitution of Infectious HCMV ∆UL50-ΣUL53

The HCMV mutant, expressing pUL53 fused to a destabilizing domain based on the cellular FKBP12 protein (ddFKBP; [55,56]), was constructed by *en passant* mutagenesis [57] using bacterial artificial chromosome (BAC)-derived HB15/AD169∆UL50 as the backbone. A fragment comprising ddFKBP together with a kanamycin resistance cassette containing all features necessary for *en passant* mutagenesis was amplified from HCMV BAC AD169-IE-FKBP-Kn (E.M.B. et al., unpublished) using primers UL53-FKBP.for (5′-GTGGACCCCACGTACGTGATAGACAAGTATGTCTAGCGTGGGAGTGCAGGTGGAAACCA-3′) and UL53-FKBP.rev (5′-GCAAGGCCGAGCGTCGTTCGCGCGGCGTGCGCACGCCGCTCAATTGGCGCGCGGATCCT-3′). The resulting PCR product was recombined with HB15/AD169∆UL50 [39], followed by excision of the KnR marker as described [57]. Correct insertion of ddFKBP into ORF-UL53 was verified by RFLP analysis of the final BAC HCMV ∆UL50-ΣUL53 and sequencing of the respective genomic region. Infectious viral particles were produced using HFF-UL50 under 500 ng/mL Dox and 1 µM Shield-1 supplementation (Hycultec GmbH, Beutelsbach, Germany) as described [39]. Collected supernatants were then transferred as infectious inoculum to fresh HFF-UL50 treated with Dox and Shield-1 for the production of a virus stock.

### 2.3. Antiviral Compounds

Antiviral compounds were derived as follows: maribavir (MedChemExpress, Monmouth Junction, NJ, USA), CDK2 Inh II (Calbiochem, Darmstadt, Germany), sotorasib (AMG510, Cay29465), neratinib (Cay18404), ibrutinib (Cay16274), osimeritinib (AZD9291, Cay16237), entacapone (Cay14153), parthenolide (Cay70080), alantolactone (Cay29762), CDDO-Me (Cay11883) (all purchased from Biomol GmbH, Hamburg, Germany) and wortmannin (12-338; Sigma-Aldrich, St. Louis, MO, USA). The investigational compounds were provided by Lead Discovery Center GmbH, Dortmund, Germany, as a series of compounds of specific interest for current developmental purposes. All these compounds are small molecules possessing the indicated molecular masses (MW range: 257.68–612.16) and are derived from various chemical classes (individual scaffolds in brackets): LDC599 (MW 411.46, scaffold quinazoline), LDC580 (485.95, quinazoline), LDC745 (454.53, diaminopyrimidine), LDC736 (285.73, quinazoline), LDC393 (386.45, benzoxazole), LDC415 (368.88, aminothiazole), LDC816 (503.47, dioxolane), LDC553 (257.68, imidazopyridine), LDC890 (401.94, imidazopyridine), LDC492 (464.54, thiazolopyrimidine), LDC279 (506.61, quinoline) and LDC266 (612.16, pyrrolopyrimidine). As a common feature, the compounds possess covalent binding properties, in most cases mediated through an acceptor of Michael addition reaction [2,29,58], which typically links the warhead to a cysteine residue of the target protein(s). Stock aliquots of all compounds were prepared in DMSO at a concentration of 10 mM.

### 2.4. Quantitative Real-Time PCR (qPCR)

The viral genome copy number in cell culture supernatants was determined by IE1-specific quantitative real-time PCR (qPCR) as described previously [59]. For viral replication kinetics and half-maximal (50%) effective concentration analyses (EC_50_) of antivirals, cells were infected with equal genome amounts and supernatants were collected at the indicated time points for proteinase K digestion and qPCR. For the qPCR reactions, aliquots of infected-cell supernatants were incubated with proteinase K (Sigma-Aldrich, St. Louis, MO, USA) at 56 °C for 1 h in order to release the viral DNA. After a denaturation step at 95 °C for 5 min, the DNA samples were optionally stored at 4 °C or directly used for qPCR, as performed in a 25 µL reaction mixture containing 5 µL of either the sample or the standard DNA solution. Additional components of the reaction mixture were 12.5 µL 2× TaqMan PCR Mastermix (Applied Biosystems, Waltham, MA, USA), 7.5 pmol of each primer (5′-AAGCGGCCTCTGATAACCAAG-3′) and 5′-GAGCAGACTCTCAGAGGATCG-3′) and 5 pmol probe directed against the HCMV MIE region exon 4 (5′-CATGCAGATCTCCTCAATGCGCGC-3′), which was labeled with 6-carboxyfluorescein reporter dye and 6-carboxytetramethylrhodamine quencher dye. The DNA standard for quantification was prepared by serial dilutions of an MIE-inserted plasmid. The thermal cycling conditions consisted of two initial steps of 2 min at 50 °C and 10 min at 95 °C followed by 40 amplification cycles (15 s at 95 °C, 1 min 60 °C). Reactions were performed with an ABI Prism 7700 sequence detector (Applied Biosystems, Waltham, MA, USA).

### 2.5. Neutral Red Assay and Fluorescence-Based Replication Assay

Cytotoxicity of the analyzed compounds was determined by the Neutral Red dye uptake assay. A total of 1.35 × 10^5^ HFFs per well were seeded in 96-well plates 1 d prior to analysis, cultivated overnight until cells were confluent and then incubated with compounds for 7 days. The assay was performed as described previously [60] using 40 μg/mL Neutral Red (Sigma Aldrich). The quantity of dye incorporation was determined using the Victor X4 microplate reader (PerkinElmer, Waltham, MA, USA) by fluorescence measurement at 560/630 nm for excitation/emission, respectively. To assess antiviral activity of compounds, HFFs seeded in 96-well plates (1.35 × 10^5^ HFFs per well) were infected with HCMV AD169-GFP or TB40-IE2-YFP to reach 25–50% infected cells at 7 days post-infection (d p.i.). After incubation with the infectious inoculum for 1.5 h at 37 °C, compounds were added in serial dilutions. At 7 d p.i., cells were fixed for 10 min at room temperature using 10% formalin. Finally, cells were washed once with PBS followed by automated fluorescence quantitation.

### 2.6. Expression Plasmids

Expression plasmids coding for the FKBP::UL53 fusion protein were generated by standard polymerase chain reaction (PCR) amplification of the respective template BAC HCMV ∆UL50-ΣUL53. After cleavage with the corresponding restriction enzymes, PCR products were inserted into the eukaryotic expression vector pcDNA3.1(+) (Life Technologies, Carlsbad, CA, USA). Expression constructs for the NanoBiT system (NanoBiT^®^ PPI, Promega, Madison, WI, USA) coding for LgBiT::UL53-Flag, LgBiT::UL53(1–87)-Flag and SmBiT::UL50-HA were generated by PCR amplification using the templates pUL53-Flag, pUL50-HA [19] and pUL53(1–87)-Flag [41]. Control plasmids were generated in an analogous manner by introducing the open reading frames coding for HCMV pUL97 (amino acids 231–280) or further viral or cellular reference proteins (pUL69, CDK7 and cyclin H) into the LgBiT/SmBiT::fusion vectors. Oligonucleotide primers used for PCR were purchased from Biomers (Table S1, Ulm, Germany). Further plasmids for the transient transfection-based expression of herpesviral NEC proteins in the confocal imaging experiments have also been described in references [19,41].

### 2.7. NanoBiT Assay for HCMV Core NEC

To assess the influence of antivirals regarding their specific activity during nuclear egress, the HCMV core NEC NanoBiT interaction system was applied. 293T cells (5 × 10^5^ cells per 6-well) were transfected with 2 µg of corresponding expression constructs (cotransfection of LgBit and SmBit constructs, or single transfection with an empty vector as background control) using PEI, as described in 2.1. At 1 to 2 d post transfection (d p.t.), biological triplicates of cells were transferred in equal confluences into a 96-well plate (white plate with clear bottom), and 3 to 4 h later, the antivirals, diluted in standard cell culture medium, were added in suitable concentrations. Directly after compound supplementation, a luciferase assay was performed using the NanoGlo Live Cell Assay System, according to the manufacturer’s protocol (N2011, Promega, Madison, WI, USA). The luciferase activity was measured at 37 °C for 1.5 h using the Victor X4 microplate reader. Background activity of LgBit luciferase activity without the SmBit counterpart was subtracted from LgBit/SmBit cotransfected cells to obtain PPI-specific luciferase activity.

### 2.8. Coimmunoprecipitation (CoIP)

For CoIP analysis, 293T cells were seeded in 10 cm dishes with a density of 5 × 10^6^ cells and used for transient transfection with expression plasmids. Then, 2–3 days post-transfection (d p.t.), CoIP was performed as described previously [61], using mAb-HA (H9658, Sigma-Aldrich, St. Louis, MO, USA). In brief, cells were lysed in 500 μL CoIP buffer (50 mM Tris/HCl (pH 8.0), 150 mM NaCl, 5 mM EDTA, 0.5% NP-40, 1 mM PMSF, 2 μg/mL aprotinin, 2 μg/mL leupeptin and 2 μg/mL pepstatin). Subsequently, total lysates were incubated with antibody-coated (2 μL of tag-specific or control antibodies) Dynabeads^®^ Protein A (30 μL per sample; Life Technologies, Carlsbad, CA, USA) for 2 h at 4 °C under rotation. The precipitates were washed five times with 1 mL of CoIP buffer. CoIP samples and lysate controls were taken prior to the addition of the CoIP antibody. SDS-PAGE and standard Western blot analysis of cell lysates was performed using equal protein amounts as described previously [62]. Antibodies used for staining were mAb-HA (H9658, Sigma-Aldrich, St. Louis, MO, USA), pAb-Flag (F7425, Sigma-Aldrich, St. Louis, MO, USA), pAb-FKBP12 (ab2918, Abcam, Cambridge, UK) and mAb-β-actin (A5441, Sigma-Aldrich, St. Louis, MO, USA).

### 2.9. Indirect Immunofluorescence (IF) Analysis and Confocal Laser-Scanning Microscopy

HFF or HFF-UL50 cells, either Dox-induced or uninduced, were seeded in 6-well plates with a density of 2 × 10^5^ cells per well, grown on coverslips and infected with HCMV AD169 or AD169 ∆UL50-ΣUL53. For infection experiments with the recombinant virus, different Shield-1 and Dox conditions were analyzed. Antivirals were added post-infection as described for the experiments. For transfection experiments, HeLa cells were seeded on coverslips in 6-well plates with a density of 3.25 × 10^5^ cells per well for cotransfection with the indicated NEC expression plasmids (HSV-1 HA-UL34 + Flag-UL31, HCMV pUL50-HA + pUL53-Flag and EBV HA-BFRF1 + Flag-BFLF2). At indicated time points after virus infection, or 3 days post-transfection, cells were fixed, applied for IF staining as described previously [45] and analyzed using a TCS SP5 confocal laser-scanning microscope with Leica LAS AF software (Leica Microsystems, Wetzlar, Germany). The microscope was utilized with a HCX PL APO lambda blue 63x/NA 1.4 OIL objective, a 405 UV laser diode, an Argon laser, a 543 HeNe laser and a 633 HeNe laser, using monochrome filters with spectral ranges of 415–477, 496–540, 553–618 and 643–709, respectively. The device features a Leica photomultiplier tube (PMT) and a hybrid detector (HyD). Images were further processed using Photoshop CS5 (Adobe Inc., San José, CA, USA). Primary and secondary antibodies used for staining were mAb-UL53.01 (kindly provided by S. Jonjic and T. Lenac Rovis, Rijeka, Croatia), mAb-lamin A/C (ab108595, Abcam, Cambridge, UK), mAb-HA (Clone 7, H9658, Sigma-Aldrich, St. Louis, MO, USA), pAb-Flag (F7425, Sigma-Aldrich, St. Louis, MO, USA), anti-mouse Alexa 555 (A21422, Thermo Fisher Scientific, Waltham, MA, USA) and anti-rabbit Alexa 488 (A11034, Thermo Fisher Scientific, Waltham, MA, USA). Microscopic counting was performed for quantitation of colocalization patterns of two different proteins, as achieved by counting 50 cells per biological triplicate. Three patterns of pUL53 localization were distinguished, as indicated in the figure legends.

## 3. Results and Discussion

### 3.1. Antiviral Screening Using an Investigational Group of Covalently Binding Warheads

**Figure 1:**
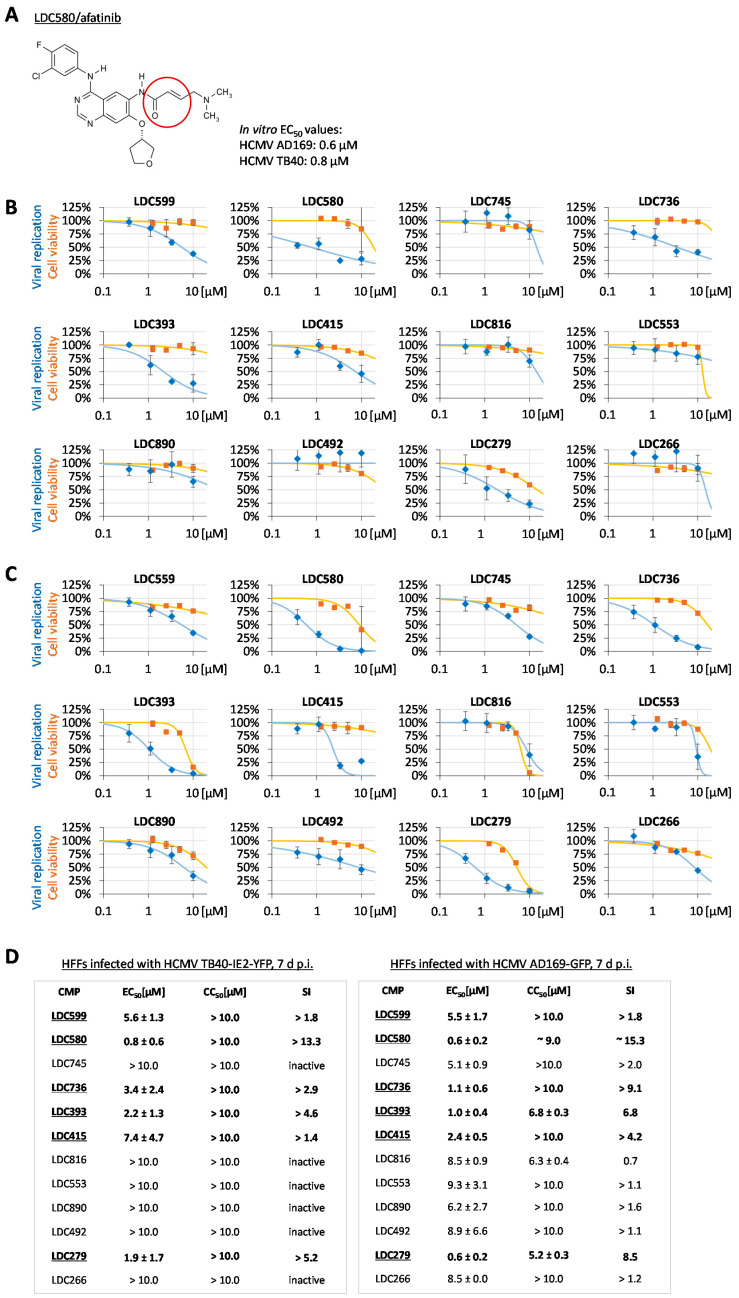
Anti-HCMV activity of investigational warhead compounds, including the clinical prototype afatinib. (**A**) Chemical structure of a clinically approved prototype of covalently binding warhead inhibitors, LDC580, afatinib/Gilotrif^®^. The red circle identifies a functionally active α-, β-unsaturated carbonyl group. (**B**) At 7 d p.i., compounds were analyzed for their cytotoxicity (orange) and antiviral activity (blue) during HCMV infection, using TB40-IE2-YFP as a reporter virus. Cell viability was assessed through Neutral Red staining of uninfected cells. Measurements were performed in quadruplicate (YFP fluorometry) or triplicate (Neutral Red assay), and standard deviations of mean values are given; EC_50_, CC_50_ and SI values are indicated accordingly. (**C**) In a parallel series of analyses, HCMV AD169-GFP was used as another reporter virus representing a second viral strain. The conditions of the antiviral assessment were identical to those for panel B. (**D**) Comparison of compounds analyzed against the two strains of HCMV: hit compounds with antiviral activity against both viral strains are marked as underlined in bold.

Seeking for a novel group of potential antiviral agents, small molecules with covalent protein-binding activity were investigated. These small molecules, originally developed as kinase inhibitors, carry a so-called warhead moiety as an important functional group, such as the prototype afatinib (Figure 1A, red circle). In this study, a series of newly generated warhead compounds (most of which are the property of B.K./Lead Discovery Center, Dortmund, Germany) were used for an evaluation of putative antiviral and core NEC-inhibitory properties. In a first step, the antiviral activity of warheads was analyzed against HCMV TB40-IE2-YFP (Figure 1B) and AD169-GFP (Figure 1C). To this end, HFFs were infected with either of the two viral strains before the GFP or YFP reporter signals were measured at 7 d p.i. (in parallel, putative compound cytotoxicity was determined by Neutral Red assay). The HCMVs AD169-GFP and TB40-IE2-YFP, based on BAC-derived recombinants of the laboratory strains AD169 and TB40, respectively, were both propagated on HFFs, and the produced stocks were then used as two reporter viruses for the assessement of antiviral drug activity. While strain AD169 is mainly restricted in its tropism to fibroblasts, TB40 tropism can be extended to fibroblasts and epithelial and endothelial cells [63,64]. Here, the use of two strains was intended to illustrate the assumption that antiviral activity of these warhead compounds may be directed to conserved viral target proteins, thus pointing out a minor impact of strains. Thus, the antiviral half-maximal effective concentrations (EC_50_ values), cytotoxic concentrations (CC_50_) and selectivity indices CC_50_/EC_50_ (SI) were determined for each compound on both viral strains (Figure 1D). For the compounds LDC599, LDC580, LDC736, LDC393, LDC415 and LDC279, a pronounced antiviral activity was detected, as indicated by the EC_50_ and SI values for TB40-IE2-YFP (Figure 1D, left) and AD169-GFP (right, hit compounds marked as underlined in bold). Here, EC_50_ values ranged between 0.6 µM and 7.4 µM, while other compounds were lower in activity, with EC_50_ values between 5.1 µM and 9.3 µM (AD169-GFP) or >10 µM (TB40-IE2-YFP). Taken together, six of the twelve analyzed warheads showed pronounced anti-HCMV activity as measured for the two viral strains.

### 3.2. A Novel Approach That Aims at the Targeting of Covalently Binding Warheads to the Viral Nuclear Egress Complex

**Figure 2:**
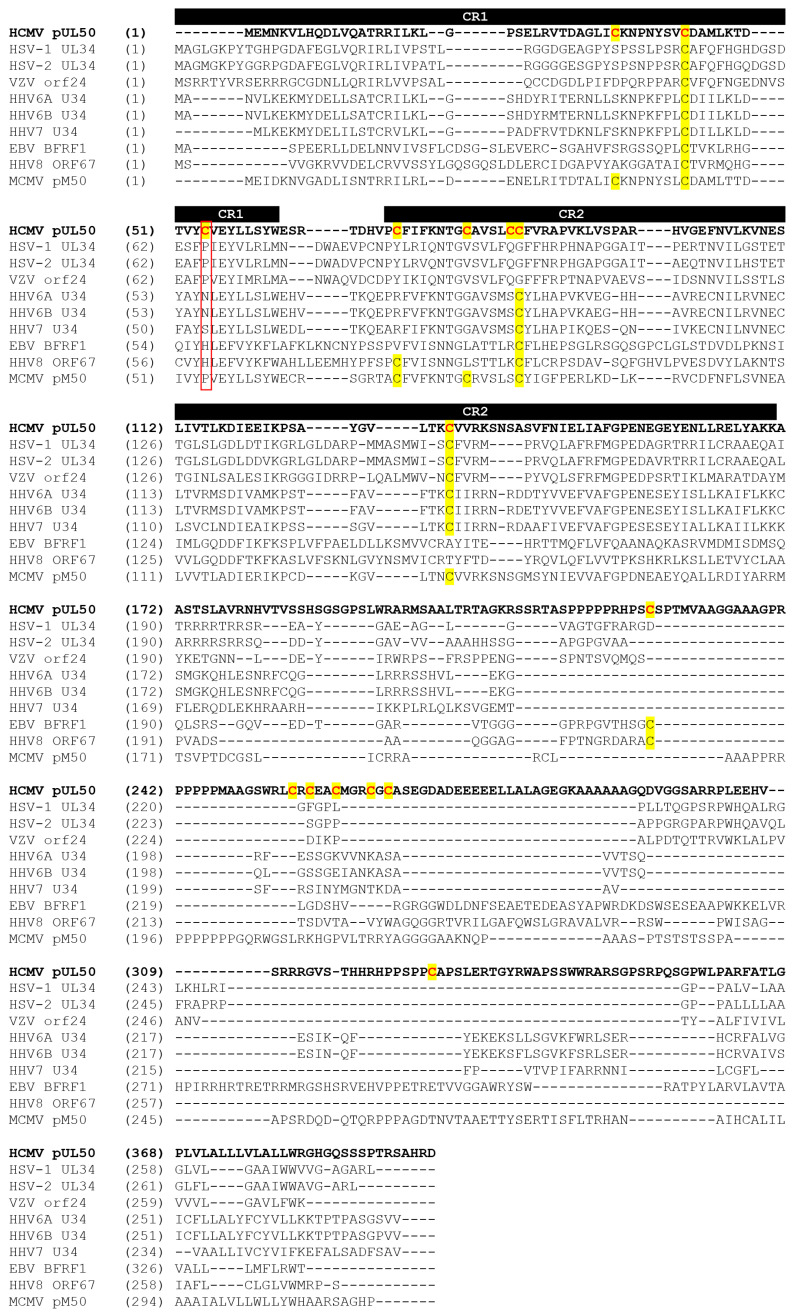
Protein sequence alignment of HCMV pUL50 and its homologs. The multiple sequence alignment was generated using Clustal Omega [65] (https://www.ebi.ac.uk/Tools/msa/clustalo/; accessed on 13 March 2023) and shows the full amino acid sequences of human cytomegalovirus (HCMV) pUL50 (UniProtKB entry: P16791), homologs of human herpesviruses (herpes simplex virus types 1 and 2 (HSV-1 UL34: P10218 and HSV-2 UL34: P89457)), varicella-zoster virus (VZV orf24: P09280), human herpesvirus 6 variants A and B (HHV-6A U34: P52465 and HHV-6B U34: Q9QJ35), human herpesvirus 7 (HHV-7 U34: P52466), Epstein–Barr virus (EBV BFRF1: P03185), human herpesvirus 8 (HHV-8/KSHV ORF67: Q76RF3) and the homolog of the murine cytomegalovirus (MCMV pM50: H2A365). Alignment coloring scheme: red on yellow, cysteine residues in the HCMV pUL50 sequence (red frame marks the uniqueness of the structurally exposed Cys54 residue of HCMV pUL50 compared to homologs); black on yellow, evolutionarily conserved cysteine residues in homologs of HCMV pUL50. Note that the N-terminal region of pUL50 (amino acids 10–169) is responsible for pUL53 binding, since this contains the previously identified conserved regions CR1 (1–62) and CR2 (70–170) [66]. The CR1 and CR2 regions are depicted on top of the sequence alignment as black bars.

The herpesviral nuclear egress complex (NEC) represents a functionally highly important determinant of viral replication efficiency. Recent studies on HCMV indicated that the disruption of the hook-into-groove interaction of pUL50 and pUL53 inhibits the late phase of viral replication [24,34,40,46]. Considering the features of core NEC sequences and structures, we addressed the question of whether distinct cysteine residues might be accessible to the attack of inhibitory warhead compounds. To this end, valuable information has been provided by the crystallization-based analyses of three different herpesviral NECs and by the specific investigation of NEC properties in terms of sequence–structure relationships [11,41,42,43]. Concerning the NEC primary sequences, eight cysteine residues were found to be present in the N-terminal, conserved regions CR1 and CR2 that define the α-helical NEC groove element of HCMV pUL50 and homologs (Figure 2) [43]. Among these, two are exclusive to HCMV (Cys54 and Cys84), while six are also present in the murine CMV homolog pM50 or additionally in further herpesviral NEC groove proteins (Cys35, Cys43, Cys71, Cys79, Cys85 and Cys133).

**Figure 3:**
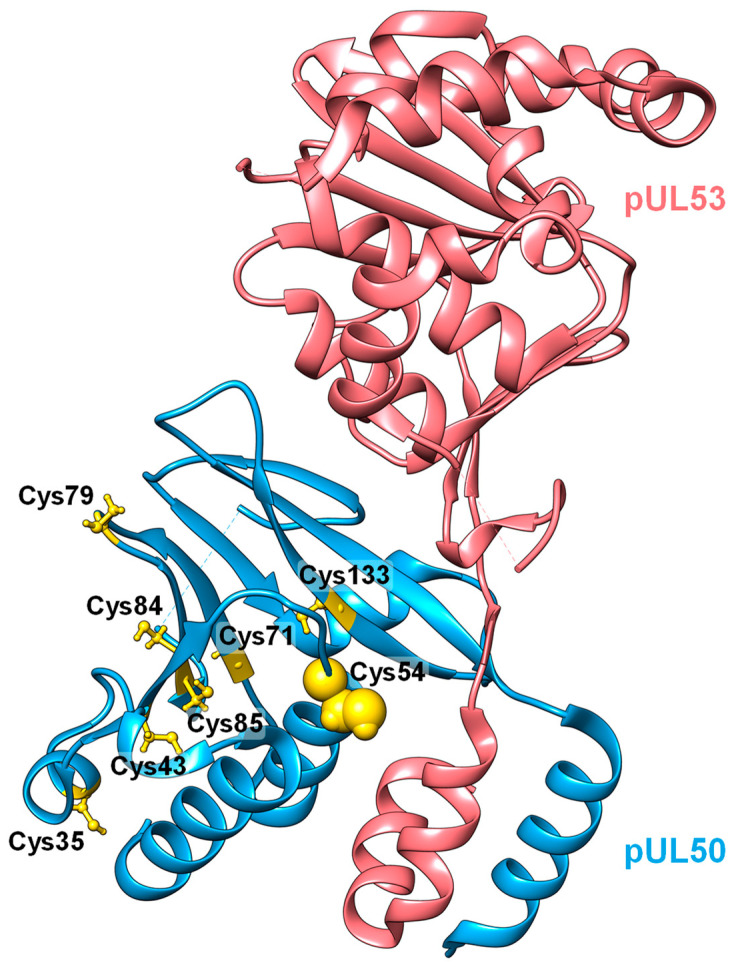
Protein complex structure of pUL53 and pUL50 from HCMV (PDB ID code: 5D5N [43]). HCMV pUL53 (red) interacts with pUL50 (blue) via a hook-like element that binds into an α-helical groove on pUL50. Cysteine residues in pUL50 are depicted with a yellow ball-and-stick representation, while Cys54, which is the only one of the eight cysteines located at the interaction interface with pUL53, is highlighted with spheres. Protein visualization was performed with UCSF Chimera [67] (version 1.16).

When considering the structural exposure of these cysteine residues, it can immediately be recognized that Cys54 is located within the center of the hook-into-groove binding region, which connects the α-helices α1, α2, α3 and α4 of pUL50 with those termed αN and αC of pUL53 [41]. In contrast to Cys54, all other locally relevant cysteine residues were found not exposed to this binding interface (Figure 3). Importantly, this residue of Cys54 has no correlate in all other herpesviral NECs investigated (see red frame, Figure 2). Thus, the information derived from the crystal structures strongly points to the accessibility of at least one cysteine residue of the HCMV NEC groove protein, and the information on primary sequences indicates the HCMV specificity of this residue. It should additionally be mentioned that no cysteine residue was found within the hook element (amino acids 55–87) of HCMV pUL53.

### 3.3. Evaluation of NEC Interaction Properties under Treatment with Primary Hits Obtained from the Group of Investigational Warheads

**Figure 4:**
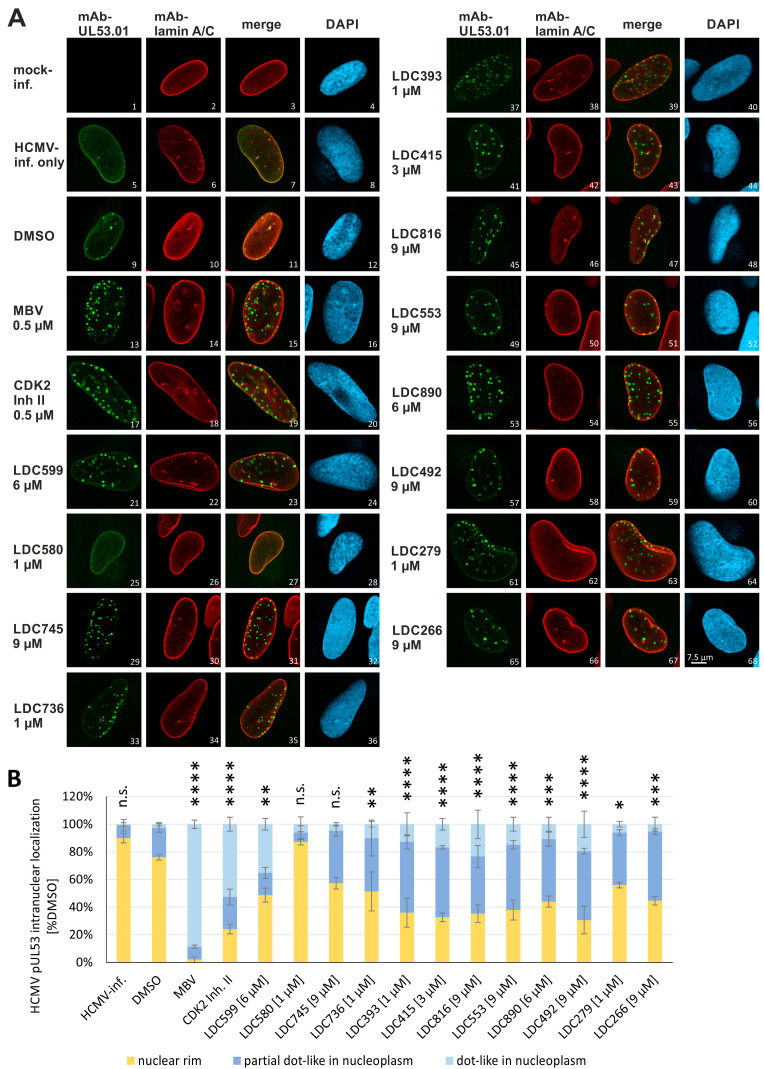
Impact of those warhead compounds possessing antiviral activity on the localization of HCMV pUL53. (**A**) HFFs were infected with HCMV AD169 (MOI 0.1) and immediately treated with warhead compounds. Compounds were applied, as based on their individual EC_50_ values, at concentrations exerting partial-grade antiviral activity (see indicated), and the determination was performed in biological triplicates. At 5 d p.i., cells were fixed, used for IF staining with the indicated antibodies and analyzed for intranuclear localization by confocal imaging. DAPI counterstaining indicated the nuclei morphology of the respective cells. Additional single channel images in grayscale, allowing easier comparison of signal patterns, are presented in Figure S1. For raw data, see https://doi.org/10.5281/zenodo.7794233; accessed on 3 April 2023 (**B**) Quantitation of this IF analysis was achieved by counting 50 cells per biological triplicate. Three patterns of pUL53 localization were distinguished, i.e., normal nuclear rim (yellow), partial dot-like intranucleoplasmic aggregation (dark blue) and predominant dot-like aggregation (light blue). Mean values ± SDs are given. Statistical analysis was performed using ordinary one-way ANOVA and post hoc Tukey testing on combined dot-like pUL53 localization values of DMSO in relation to the analyzed ibrutinib concentrations and MBV; * *p* ≤ 0.05, ** *p* ≤ 0.01, *** *p* ≤ 0.001, **** *p* ≤ 0.0001; n.s., not significant.

In order to address the question whether these compounds have a measurable impact on the PPI and intracellular localization of core NEC proteins, first, a confocal imaging-based analysis was performed. To this end, HFFs were infected with HCMV AD169 and treated with the warheads at concentrations referring to partial-grade antiviral activity (based on individual EC_50_ values). At 5 d p.i., the typical nuclear rim localization of pUL53 was analyzed by indirect immunofluorescence staining and confocal imaging (Figure 4A). In addition, lamin A/C was stained as a marker of the nuclear rim. The compounds MBV and CDK2 Inh II were used as positive controls for the disruption of the rim localization of pUL53 [24], resulting in a dot-like structure of pUL53 (images 13 and 17). The investigated warhead compounds showed individually different impacts on pUL53 localization. Some compounds resulted in the accumulation of distinct dot-like, punctate structures of pUL53 in the nucleoplasm (e.g., images 21, 45 and 53), while compound LDC580 showed no comparable effect (image 25). Interestingly, several of the compounds additionally produced some slight degree of intranucleoplasmic coaggregation of lamin A/C (in particular, LDC599, LDC745, LDC736 and LDC266). This effect was also visible for the kinase reference inhibitors MBV and CDK2 Inh II (Figure 4A, images 14 and 18), so that, in addition to direct NEC targeting, an involvement of inhibitory kinase targeting by these LDC warheads might be suggestive. Since part of these lamin A/C signals, however, could also be found in DMSO control panels (Figure 4A, image 10), albeit at lower numbers, this observation remains preliminary at this stage. Importantly, the microscopic analysis was quantified by counting 50 cells per biological triplicate, and the localization of pUL53 was assigned to the groups of normal nuclear rim (yellow), partial dot-like intranucleoplasmic aggregation (dark blue) and predominant dot-like aggregation (light blue) (Figure 4B). In contrast to the strict nuclear rim localization of pUL53 in HCMV-infected cells, MBV or CDK2 Inh II treatment resulted in predominant dot-like structures. Moreover, the quantitation demonstrated that compounds LDC599, LDC393, LDC415, LDC816, LDC553, LDC890, LC492 and LDC266 appeared to induce a partial or predominant dot-like localization of pUL53 within the nucleoplasm in more than 50% of the cells. It should be mentioned, however, that this analysis based on confocal imaging, i.e., providing indirect evidence of NEC interaction in cells, cannot fully exclude the possibility of additional secondary target effects of the compounds. Nonetheless, three of the thirteen analyzed warheads (LDC599, LDC393 and LDC415) were considered as hit compounds showing effects in both readouts, i.e., an inhibitory impact on viral replication (EC_50_) and on the pUL53 localization (dot-like aggregation).

**Figure 5:**
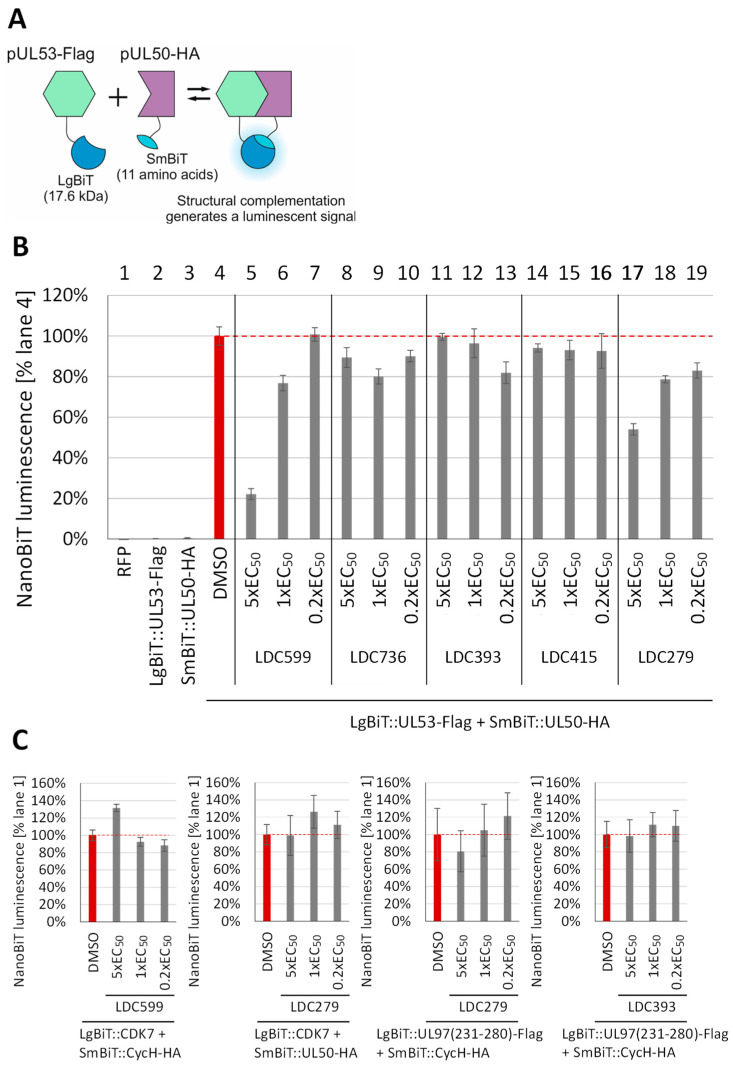
Inhibitory impact of selected LDC warheads on the HCMV core NEC interaction. (**A**) Schematic illustration of the cloned fusion constructs expressing one part (LgBiT or SmBiT) of the split luciferase NanoLuc. SmBiT was fused to pUL50-HA and LgBiT to pUL53-Flag. Note that interaction of the core NEC heterodimer led to structural complementation of the luciferase, which generated a measurable luminescent signal. (**B**) 293T cells were transiently transfected with these constructs for assaying in the NanoBiT system. At 1 d p.t., biological triplicates of cells were transferred into a 96-well plate. Compound treatment was started directly before NanoGlo Live Cell Reagent addition, and compound concentrations referring to 5×, 1× or 0.2× anti-HCMV EC_50_ levels were applied. After addition of the NanoGlo Live Cell Reagent, measurements were immediately performed to quantify luminescence for 2 h. Each of the constructs, expressed separately together with empty vectors, served as controls of background signals, and RFP served as a transfection control. Mean values ± SDs are given. (**C**) Serving as a specificity control, additional pairs of NanoBiT test constructs were used, as these were not supposed to be targeted by the analyzed LDC compounds (i.e., LgBiT::CDK7 + SmBiT::CycH-HA, LgBiT::CDK7 + SmBiT::UL50-HA and LgBiT::UL97(231–280)-Flag + SmBiT::CycH-HA; see Table S1 and Section 2.6 for details of plasmid construction). Note the lack of concentration-dependent inhibitory impact of LDC599 (strongly active against viral pUL50–pUL53, LDC279 (moderately active) and LDC393 (inactive) as based on signal determination by the NanoBiT system. Mean values ± SDs of biological triplicates are given.

Moreover, a NanoBiT analysis was performed to confirm the inhibitory activity of these compounds towards viral core NEC interaction. For this purpose, a NanoBiT system was established, in which the two components LgBiT and SmBiT of a split luciferase (NanoLuc) were fused to the core NEC protein-coding sequences of pUL53 or pUL50. These fusion constructs were cloned into expression vectors to generate SmBiT::UL50-HA and LgBiT::UL53-Flag. By the interaction of pUL50 with pUL53, the luciferase was structurally complemented, resulting in a measurable luminescent signal (Figure 5A). The quantitation of PPI between LgBiT::UL53-Flag and SmBiT::UL50-HA provided the posititive control, defined as 100%. The most active LDC warhead compounds, i.e., LDC599, LDC736, LDC393, LDC415 and LDC279, were thus selected for an analysis of core NEC inhibitory activity (Figure 5B). The data of this NanoBiT analysis revealed a strong concentration-dependent inhibitory effect for LDC599, a partial effect for LDC279 and basically no effect for the other three compounds. For ascertaining the specificity of reactions, additional pairs of NanoBiT test constructs were used as negative controls, as these were not supposed to be targeted by the analyzed LDC compounds, i.e., LgBiT::CDK7 + SmBiT::CycH-HA, LgBiT::CDK7 + SmBiT::UL50-HA and LgBiT::UL97(231–280)-Flag + SmBiT::CycH-HA (Figure 5C). To this end, three compounds were selected on the basis of their showing inhibitory activity of pUL50–pUL53 interaction in the NanoBiT system (see Figure 5B), namely, LDC599 (strongly active), LDC279 (moderately active) and LDC393 (inactive). As a relevant finding regarding these controls, none of the three LDC compounds showed a substantial, concentration-dependent inhibitory effect on the selected reference constructs. Thus, two of this series of investigational LDC warheads, LDC599 and LDC279, exerted a blocking activity towards the pUL50–pUL53 interaction in this reporter system. Lack of activity by other warheads, which actually showed an effect on the nuclear rim localization of these proteins by confocal imaging-based evaluation, may be explained by additional factors that can likewise have impacts on viral nuclear rim formation, such as core NEC oligomerization and phosphorylation/kinase-specific effects, as described before [24,25,32,40]. Combined, these results are compatible with the concept that the antiviral activity of these warheads is linked to interference with the core NEC nuclear rim localization.

### 3.4. Clinically Approved and Developmental Warhead Drugs: Initial Assessment of Antiviral Activity

**Figure 6:**
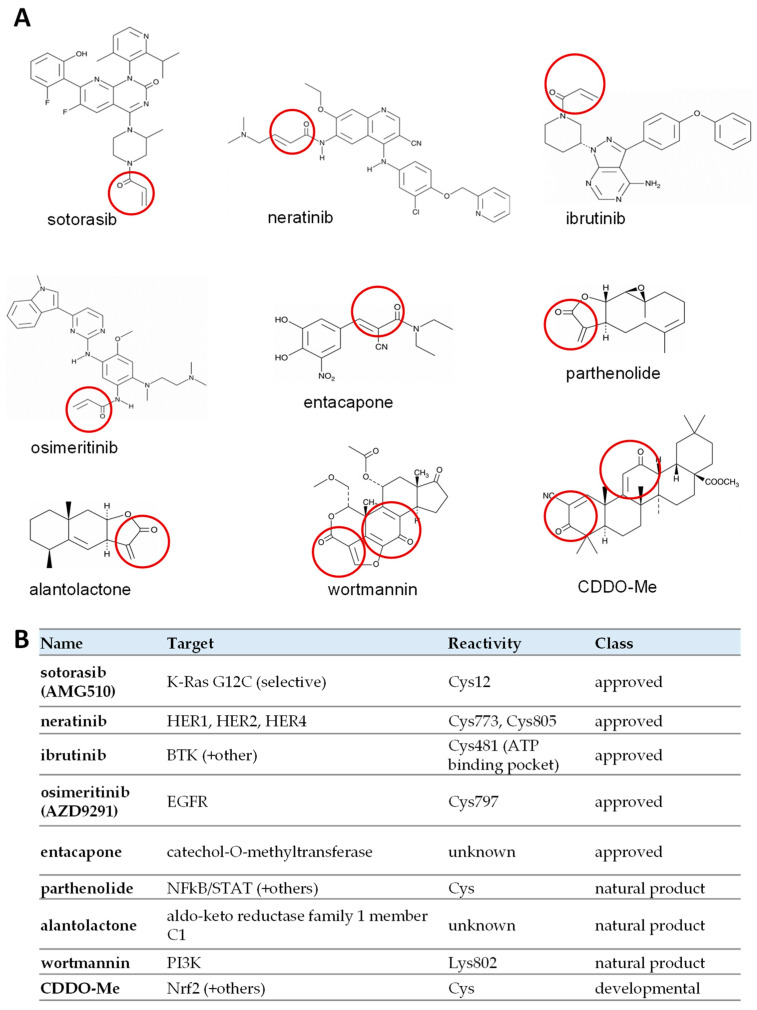
Chemical structures and properties of currently available clinically relevant warheads. (**A**) Chemical structures of several warheads commercially available. Red circles identify functionally active α-, β-unsaturated carbonyl groups. CDDO-Me = 2-cyano-3,12-dioxooleana-1,9(11)-dien-28-oic acid methyl ester. (**B**) Overview of primary biological targets, the known targeted amino acids and drug classes (admission status). Sources: sotorasib [68], neratinib [58,69], ibrutinib [70], osimeritinib [71], entacapone [72], parthenolide [58,73], alantolactone [58,74], wortmannin [58,75], CDDO-Me [58,76].

To further investigate the inhibitory potential of covalent binders, a selection of commercially available, clinically relevant warheads were analyzed. The majority of these compounds represented developmental or approved drugs in clinical use. Furthermore, three natural substances were added, for which exact reactivities have not yet been identified. The selected compounds contained at least one α-, β-unsaturated carbonyl group, i.e., a functionally active warhead (Figure 6A, highlighted in red). Notably, the covalent binding properties of warheads may generally involve secondary targets. Available information about the primary biological targets, the known targeted amino acids and the drug classes (admission status) is summarized in Figure 6B.

**Figure 7:**
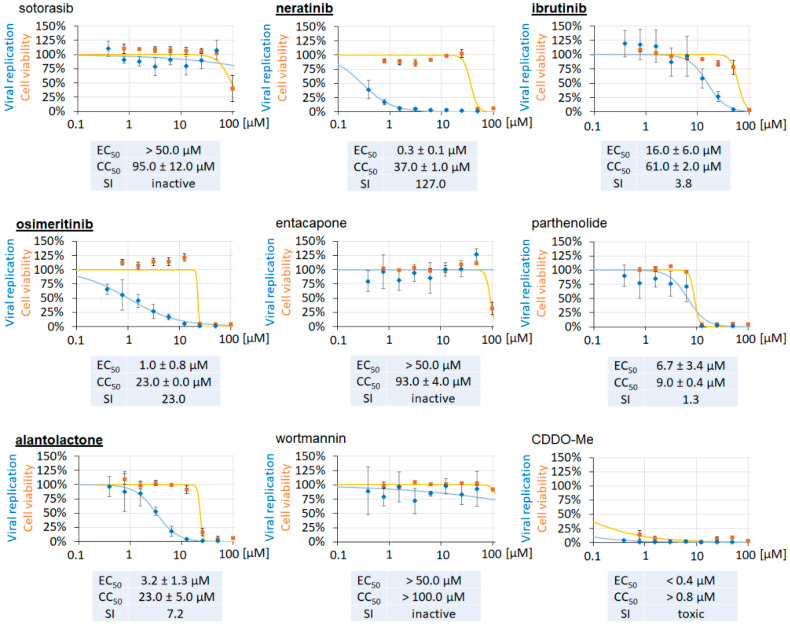
Antiviral activity of clinically relevant warhead compounds. HCMV AD169-GFP-infected HFFs were treated with compounds to analyze their antiviral activity via quantitation of the GFP reporter signal at 7 d p.i. In parallel, cell viability was assessed using Neutral Red staining of uninfected, compound-treated cells. Mean values ± SDs are given; EC_50_, CC_50_ and SI values are indicated below the curves. Hit compounds are marked as underlined in bold.

First, the selected compounds were evaluated regarding their antiviral activity and cytotoxicity (Figure 7). HCMV AD169-GFP-infected HFFs were treated with 0.1 µM to 50 µM of the compounds (in parallel Neutral Red assay, 0.1 µM to 100 µM), before the signal assessments were performed at 7 d p.i. Antiviral activity was measured in a concentration-dependent manner (Figure 7, blue curves), and prioritized compounds were selected on the basis of their lack of cytotoxicity (orange curves). Four compounds, i.e., neratinib, ibrutinib, osimeritinib and alantolactone, were classified as promising candidates (marked as underlined in bold). Their selecticity indices (SI values) indicated concentration ranges that comprised true anti-HCMV activity in the absence of cytotoxicity, so that these four compounds were selected for further investigations. In contrast, for sotorasib, entacapone, parthenolide, wortmannin and CDDO-Me, no notable antiviral activity could be determined.

### 3.5. Evaluation of NEC Interaction Properties under Treatment with the Clinically Relevant Warheads

**Figure 8:**
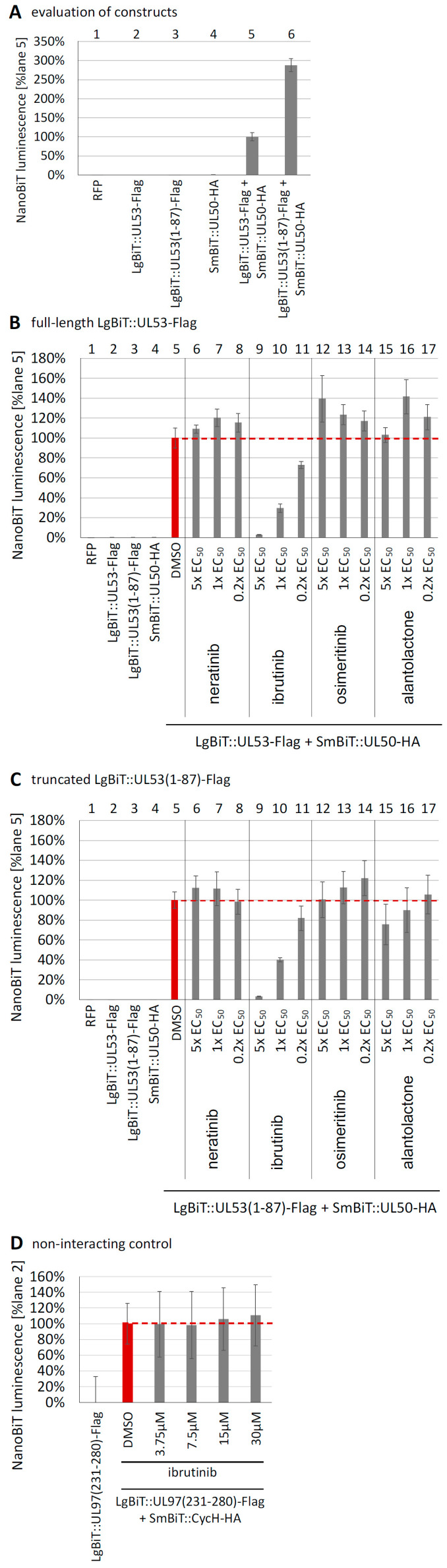
Inhibitory impact of hit warheads on the HCMV core NEC interaction. (**A**) 293T cells were transiently transfected with the constructs LgBiT::UL53-Flag, LgBiT::UL53(1–87)-Flag or SmBiT::UL50-HA for assaying in the NanoBiT system. At 1 d p.t., biological triplicates of cells were transferred into a 96-well plate. After addition of NanoGlo Live Cell Reagent, measurements were immediately performed to quantify luminescence for 2 h. Each of the constructs, expressed separately together with empty vectors, served as controls of background signals, and RFP served as a transfection control. In (**B**), the construct LgBiT::UL53-Flag served as the primary binding component, while in (**C**) the truncated construct LgBiT::UL53(1–87)-Flag was alternatively applied. Assay conditions were identical to those for panel A. Compound treatment was started directly before NanoGlo Live Cell Reagent addition, and compound concentrations referring to 5×, 1× or 0.2× anti-HCMV EC_50_ levels were applied. (**D**) As a control, which was not supposed to interact with the drug ibrutinib, viral pUL97 interaction with cyclin H was used in the respective pair of test constructs (LgBiT::UL97(231–280)-Flag + SmBiT::CycH-HA). Note the lack of concentration-dependent inhibitory impact of ibrutinib on this pUL97–cycH interaction signal. Mean values ± SDs are given.

To investigate whether the clinically relevant compounds may interfere with core NEC protein–protein interactions, another NanoBiT measurement was performed. In this case, two core NEC protein-coding sequence versions of pUL53 were fused to LgBiT, either in full length or truncated to the interaction region of pUL53(1–87). This led to the generation of the constructs SmBiT::UL50-HA, LgBiT::UL53-Flag and LgBiT::UL53(1–87)-Flag, which were confirmed as functionally active (Figure 8A). Interestingly, PPI of the truncated version LgBiT::UL53(1–87)-Flag with SmBiT::UL50-HA resulted in a strongly increased signal strength of 288%. This increase in binding affinity might be due to LgBiT fusion to the isolated pUL53 hook element of amino acids 1–87, which has been identified as the main determinant of core NEC heterodimeric interaction by previous studies [11,41,43]. In further experimentation, the posititive control pairs, LgBiT::UL53-Flag and SmBiT::UL50-HA (Figure 8B) or LgBiT::UL53(1–87)-Flag and SmBiT::UL50-HA (Figure 8C), were used as references defining 100% of NanoBiT activity. Thereby, the four warhead drugs neratinib, ibrutinib, osimeritinib and alantolactone were applied at concentrations referring to 5×, 1× or 0.2× anti-HCMV EC_50_ levels. All compounds were analyzed in a comparative setting using either LgBiT::UL53-Flag (Figure 8B) or LgBiT::UL53(1–87)-Flag (Figure 8C). In both cases, the treatment with ibrutinib resulted in the most prominent, dose-dependent reduction in the measured interaction signal, whereas neratinib, osimeritinib and alantolactone showed very minor or no effects. To exclude the cytotoxicity of the four hit warheads in the NanoBiT-based cell system, 293T cells were separately treated with serial compound concentrations (0.78 µM to 100 µM), to be measured by Neutral Red staining at indicated time points (Figure S2). The CC_50_ of ibrutinib was 30 µM and 98 µM at 4 h and 2 d post-treatment, respectively. Thus, the effect of ibrutinib was supposed to be based on an interference with the NEC hook-into-groove interaction and seemed promising as a core NEC-directed candidate compound. In order to rule out the possibility that ibrutinib may interfere with the NanoLuc backbone, i.e., the LgBiT–SmBiT interaction of the assay system, we analyzed additional interaction pairs as a control in parallel. Here, no inhibitory effect of ibrutinib was noted on the pUL97–cyclin H interaction (amino acids 231–280, representing the cyclin H binding interface IF2 of pUL97 [77]). Thus, the high concentration range of 3.75–30 µM of ibrutinib was inactive in the case of pUL97–cyclin H (Figure 8D). This absence of drug background interference with the control LgBiT–SmBiT interaction pair appeared plausible, when considering that no cysteine was present in the concordant NanoLuc reporter. It has to be mentioned, however, that the NanoBiT system seems to have limitations in terms of defining a strict selectivity panel of drugs towards a selection of targets, at least at higher micromolar drug concentrations (Marschall et al., unpublished data). As based on our experience with the analysis of several series of warheads (including ibrutinib), directed to various target proteins, the NanoBiT system did not allow us to work out a clear monoselectivity of test compounds when comparing binding constructs, such as those used in Figure 5 and Figure 8. This finding might be explained, on the one hand, by the high sensitivity of the NanoBiT assay towards drug interference per se, or, on the other hand, by an inherent tendency of promiscuity of warhead compounds towards cysteine binding in putative targets. Particularly in the case of ibrutinib, it should be emphasized, however, that the drug represents a clinically approved drug (Figure 6B), for which primary targeting has been demonstrated to one specific cysteine residue in the Bruton’s tyrosine kinase (BTK, Cys481 [70]). Interestingly, previous studies of our group did not detect BTK association with the HCMV core NEC [21]. Instead, our recent work strongly suggested the association of cyclin-dependent kinases (CDKs; [24]), which have not been specified as ibrutinib targets. Thus, an additional kinase-based effect of the ibrutinib inhibitory activity can not be fully excluded; however, experimental evidence for this theory is missing. As far as the reported Cys481 preference for BTK targeting is concerned, pronounced promiscuity of the drug seems rather improbable. Nevertheless, secondary targets are generally possible, particularly considering the high expression levels of non-host, virus-encoded proteins, i.e., those carrying structurally exposed cysteines.

**Figure 9:**
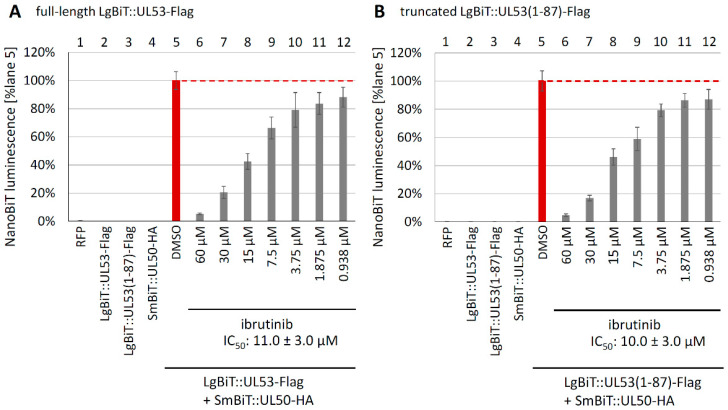
Inhibitory impact of serial concentrations of ibrutinib on core NEC interaction. The NanoBiT assay was performed analogously to procedures described in Figure 6, applying ibrutinib in a concentration range from 0.938 µM to 60 µM. In (**A**), the construct LgBiT::UL53-Flag served as the primary binding component, while in (**B**) the truncated construct LgBiT::UL53(1–87)-Flag was used.

**Figure 10:**
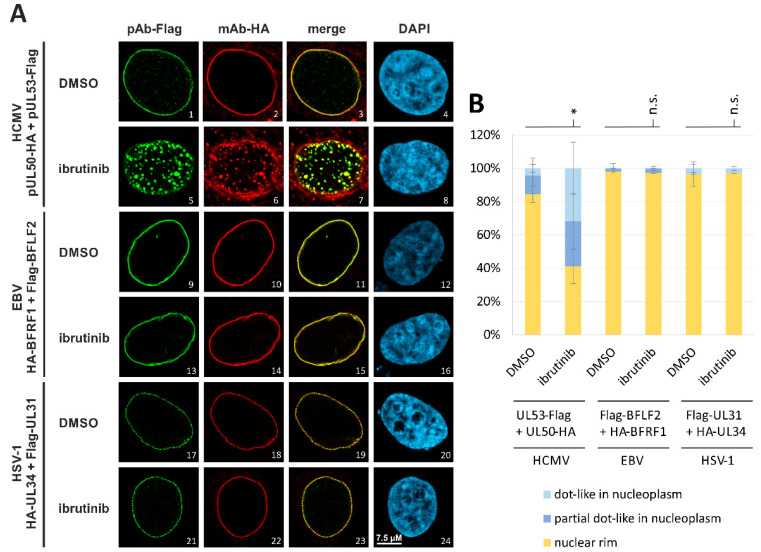
Inhibitory impact of hit compound ibrutinib on the localization of three different herpesviral core NECs in plasmid-cotransfected cells. (**A**) HeLa cells were cotransfected with each two of the indicated NEC expression plasmids (HSV-1 HA-UL34 + Flag-UL31, HCMV pUL50-HA + pUL53-Flag and EBV HA-BFRF1 + Flag-BFLF2). Ibrutinib was applied at a concentration of 4 µM immediately after transfection. At 3 d post-transfection (p.t.), cells were fixed, used for IF staining with the indicated tag antibodies and analyzed for intranuclear localization by confocal imaging. DAPI counterstaining represented the nuclear morphologies of the respective cells. Additional single channel images in grayscale, allowing easier comparison of signal patterns, are presented in Figure S4. For raw data, see https://doi.org/10.5281/zenodo.7794233, accessed on 3 April 2023. (**B**) Quantitation of this IF analysis was achieved by counting 50 cells per biological triplicate. Three patterns of pUL53 localization were distinguished, i.e., normal nuclear rim (yellow), partial dot-like intranucleoplasmic aggregation (dark blue) and predominant dot-like aggregation (light blue). Mean values ± SDs are given. Statistical analysis was performed using ordinary one-way ANOVA and post hoc Tukey testing on combined dot-like pUL53 localization values of DMSO in relation to the analyzed ibrutinib concentration and MBV; * *p* ≤ 0.05; n.s., not significant.

To further verify the result, additional concentrations of ibrutinib, ranging between 0.938 µM and 60 µM, were applied in a second setting. Here, the NanoBiT system confirmed the dose-dependent reduction in interaction, as seen for both pUL53 constructs (Figure 9A,B). The calculated IC_50_ values, defining 50% inhibition of NanoBiT interaction, were almost identical for the two constructs, i.e., IC_50_ of 11 ± 3 µM for the full length (Figure 9A) and 10 ± 3 µM for the truncated version (Figure 9B), thus providing a refinement of our concept. In essence, four of the nine clinically relevant warhead drugs showed an antiviral effect, and one of these four, ibrutinib, exerted a strong inhibition of pUL50–pUL53 interaction measurable by the NanoBiT system. Thus, based on the fact that among a total of 21 warheads investigated in this study, 9 of which were analyzed in the NanoBiT system, only 2 exerted a measurable NEC-directed activity (LDC599, Figure 4 and Figure 5B, and ibrutinib, Figure 8), at least a partial level of scaffold-mediated specificity within this group of drugs is indicated. Moreover, our results support the concept that covalent NEC binders, first of all, ibrutinib, can orincipally exert an NEC-targeted antiviral effect. In addition, further warheads (neratinib, osimeritinib and alantolactone), which likewise showed an antiviral effect but no NEC-binding activity, may act in an NEC-independent inhibitory manner, thus possibly binding to other virus-supportive targets, such as regulatory protein kinases [16,24,27,48].

In order to further address the putative NEC-blocking activity of ibrutinib, a CoIP analysis was performed in the presence or absence of the drug. Hereby, serial concentrations of ibrutinib were added to settings of plasmid-cotransfected cells (pcDNA-UL50-HA + UL53-Flag). In one case, ibrutinib was added directly to the cells at 24 h post-transfection (p.t.) (Figure S3A); in the second case, ibrutinib was added after cell lysis and was maintained during the CoIP reaction (Figure S3B). In both cases, total lysates were prepared at 48 h p.t., and the CoIP analysis was performed under continued drug treatment (Figure S3). The protein applied as the basis for IP (using tag-specific mAb-HA) was pUL50-HA, and pUL53-Flag was detected as the CoIP partner. A densitometric determination was tried, but due to the fact that protein detection via Western blotting (Wb) is generally a semi-quantitative approach, the values showed high variability, so that no statistically significant drug effect could be determined. Nevertheless, a trend of a reduction in coimmunoprecipitated pUL53-Flag under ibrutinib treatment became visible, mostly at the highest drug concentrations (Figure S3A,B, CoIP panels, lanes 20–40 µM; see in comparison lane DMSO as the solvent control). In the experiment of Figure S3A, however, the levels of transient expression of pUL50 and pUL53 were also strongly reduced under ibrutinib treatment (see lysate controls). In this setting, a nonspecific, cytotoxicity-based suppression of cellular protein levels appeared rather unlikely, since the determined drug cytotoxicity ranged at higher concentrations (CC_50_ 61.0 ± 2.0 µM; Figure 7). More likely appeared the explanation that ibrutinib already cotranslationally interfered with pUL50–pUL53 expression in the cotransfected cells, thereby inducing a destabilizing effect on both proteins by preventing their heterodimeric complexation. Such a destabilizing effect, leading to partial protein degradation, has been described for pUL50 and pUL53 before, i.e., occurring under conditions where the stabilizing heterodimeric and oligomeric pUL50–pUL53 interaction was prevented [21,44,66]. This unwarranted effect on protein levels was mostly avoided in the setting of Figure S3B, in which ibrutinib was only added after cell lysis. This condition, however, presupposed that the drug was able to resolve the already existing core NEC that had been pre-formed in cotransfected cells. Here, also, only a trend of drug-mediated inhibitory impact on the NEC became visible. Likewise, under these conditions, a reduced protein stability could not be fully excluded, i.e., considering the notion that unbound lysate fractions of pUL53 and pUL50 may be more accessible to degradation than the stabilized core NEC heterodimer. Thus, with these CoIP experiments, a partial inhibitory effect may additionally point to NEC targeting by ibrutinib, albeit no clearcut, quantitative drug-mediated NEC dissolution could be shown.

As a powerful tool of investigation, we again applied confocal imaging-based evaluation of the NEC nuclear rim localization, this time comparing different herpesviral core NECs with each other. For this purpose, pairs of α-, β- and γ-herpesviral NEC proteins were coexpressed by transient cotransfection of constructs in HeLa cells, i.e., HSV-1 HA-UL34 + Flag-UL31, HCMV pUL50-HA + pUL53-Flag and EBV HA-BFRF1 + Flag-BFLF2, respectively (Figure 10).

In all cases, ibrutinib treatment (4 µM) was performed in comparison to DMSO (solvent control). For the DMSO control samples, the confocal imaging analysis showed a complete nuclear rim signal of pronounced colocalization for the two core NEC proteins. Importantly, a marked difference in ibrutinib effects was noted for the chosen herpesviral core NECs. While HCMV pUL50–pUL53 strongly responded to the drug treatment by a predominant dot-like aggregation and speckle formation (Figure 10A, images 5–8), the two α- and γ-herpesviral NEC pairs, analyzed in parallel, did not show any similar alteration in nuclear rim localization under this drug concentration (Figure 10A, images 13–16, 21–24). The quantitative microscopic analysis confirmed the preservation of a mostly normal nuclear rim localization (yellow) under drug treatment for EBV and HSV-1 (Figure 10B, middle and right). In contrast, a significant change in the predominant dot-like aggregation (light blue) or the partial dot-like intranucleoplasmic aggregation (dark blue) of the NEC proteins was measured for HCMV (Figure 10B, left). This finding indicated that the NEC-directed ibrutinib effect of nuclear rim delocalization was specific to HCMV but did not occur in the case of the HSV-1 or EBV NEC protein homologs. Notably, our ongoing investigations also include the analysis of ibrutinib against the MCMV NEC homologs. Initial results suggest a lack of NEC rim-disturbing activity towards pM50–pM53, thus further underlining the HCMV specificity of this drug effect.

**Figure 11:**
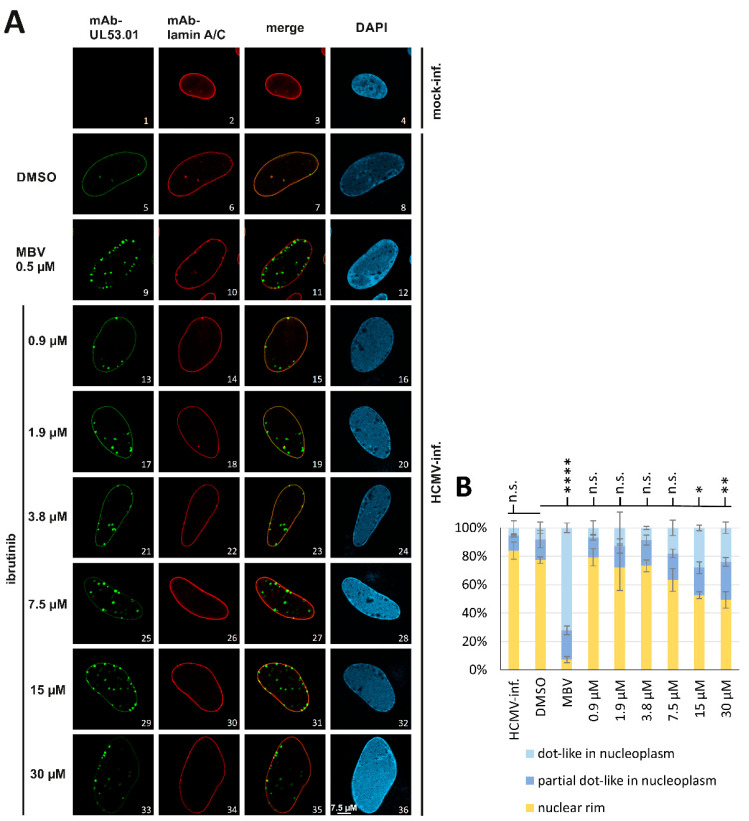
Inhibitory impact of hit compound ibrutinib on the localization of HCMV pUL53. (**A**) HFFs were infected with HCMV AD169 (MOI 0.1) and immediately treated with the warhead compound. Ibrutinib was applied at the indicated concentrations, and the determination was performed in biological triplicates. At 5 d p.i., cells were fixed, used for IF staining with the indicated antibodies and analyzed for intranuclear localization by confocal imaging. DAPI counterstaining represented the nuclear morphologies of the respective cells. Additional single channel images in grayscale, allowing easier comparison of signal patterns, are presented in Figure S5. For raw data, see https://doi.org/10.5281/zenodo.7794233 accessed on 3 April 2023. (**B**) Quantitation of this IF analysis was achieved by counting 50 cells per biological triplicate. Three patterns of pUL53 localization were distinguished, i.e., normal nuclear rim (yellow), partial dot-like intranucleoplasmic aggregation (dark blue) and predominant dot-like aggregation (light blue). Mean values ± SDs are given. Statistical analysis was performed using ordinary one-way ANOVA and post hoc Tukey testing on combined dot-like pUL53 localization values of DMSO in relation to the analyzed ibrutinib concentrations and MBV; * *p* ≤ 0.05, ** *p* ≤ 0.01, **** *p* ≤ 0.0001; n.s., not significant.

Next, a similar confocal imaging experiment was performed on the HCMV core NEC in HCMV-infected cells (Figure 11). To this end, HFFs were infected with HCMV AD169 and treated with ibrutinib at serial concentrations between 1 µM and 30 µM. At 5 d p.i., the typical nuclear rim localization of pUL53 was analyzed by indirect immunofluorescence staining and confocal imaging (Figure 11A). This analysis also visualized an accumulation of distinct dot-like structures of pUL53 (e.g., images 25, 29 and 33) as increasing in distinctness under rising concentrations of the drug. Interestingly, no additional intranucleoplasmic coaggregation of lamin A/C was detected in the case of ibrutinib, which was different to the findings decribed for LDC warhead compounds depicted in Figure 4. This might indicate a more specific targeting of ibrutinib to the viral core NEC, i.e., to the pUL53 localization behavior, than was found for other compounds, but this hypothesis awaits further confirmation. The result was supported by microscopic counting to achieve a quantitative signal evaluation (Figure 11B), thus demonstrating a statistically significant drug-mediated distortion of the viral NEC nuclear rim formation. As a methodological trait, however, it has to be emphasized that the speckled appearance of nuclear rim, i.e., the drug-mediated disruption of the typical NEC nuclear rim localization, does not allow the direct conclusion of a dissociation of the pUL50–pUL53 core NEC heterodimer/oligomer. Previous studies of our group demonstrated that the speckled appearance of the HCMV core NEC can be induced by both direct inhibitors of core NEC proteins and indirect inhibitors of NEC-associated proteins [24,25,34,40]. As an important finding of the present study, nonetheless, this novel group of warhead small molecules likewise exerts a pronounced phenotypic effect on the viral NEC nuclear rim localization. Specifically for ibrutinib, the findings support our statement of a warhead- exerted mode of action that is, at least in part, directed to the HCMV-specific core NEC.

### 3.6. A Novel Experimental System, Based on Conditionally Regulated Core NEC Expression of Recombinant HCMV ΔUL50-ΣUL53, for the Assessment of Core NEC-Directed Inhibitors

**Figure 12:**
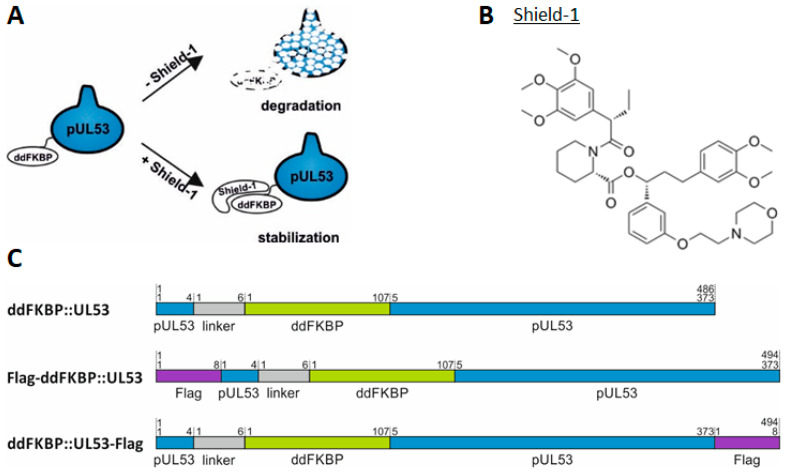
Principle of the Shield-1-regulated protein stability through fusion with a destabilizing domain. (**A**) Schematic illustration of the regulatory system, in which pUL53 is fused to an unstable variant of the FK506-binding protein FKBP (ddFKBP) that is stabilized by interaction with the synthetic ligand Shield-1. (**B**) Chemical structure of the stabilizing ligand Shield-1 containing a morpholine group that enhances its intracellular availability [55]. (**C**) Schematic illustration of ddFKBP::UL53 fusion constructs: pUL53, blue; linker region GSARQL, gray; ddFKBP, green; Flag-tag, purple; amino acid positions are represented by numbers.

The approaches hitherto used in the study, i.e., antiviral assays, confocal imaging, NanoBiT and CoIP, provided a collection of data suggesting, at least in part, an inhibitory effect of warhead compounds on the viral core NEC. However, a more sophisticated system of conditional expression of pUL50 and/or pUL53 in the context of HCMV replication was established to specify such statements. To this end, a recombinant HCMV was generated for the investigation of core NEC inhibitors and other NEC-specific functional aspects. This viral construct was based on the BAC HCMV AD169 ΔUL50 [39], in which ORF-UL50 had been deleted. This deletion could be complemented by a cell population with Dox-inducible overexpression of HCMV pUL50 (HFF-UL50). In the present study, pUL53, expressed from the viral genome, was additionally modified by fusion to a destabilizing domain (ddFKBP), as linked through a short spacer region of six amino acids (Figure 12A). In the absence of the specific ligand, Shield-1, pUL53 should be degraded, whereas the addition of Shield-1 should stabilize the fusion protein ddFKBP::UL53 (Figure 12B,C). In fact, this system led to a conditionally regulated expression of pUL53. When combining the two options of conditional expression, i.e., ΔUL50 and ddFKBP::UL53, a recombinant virus termed HCMV ΔUL50-ΣUL53 should provide a multifaceted tool for the analysis of the HCMV core NEC regulation and NEC inhibitors.

**Figure 13:**
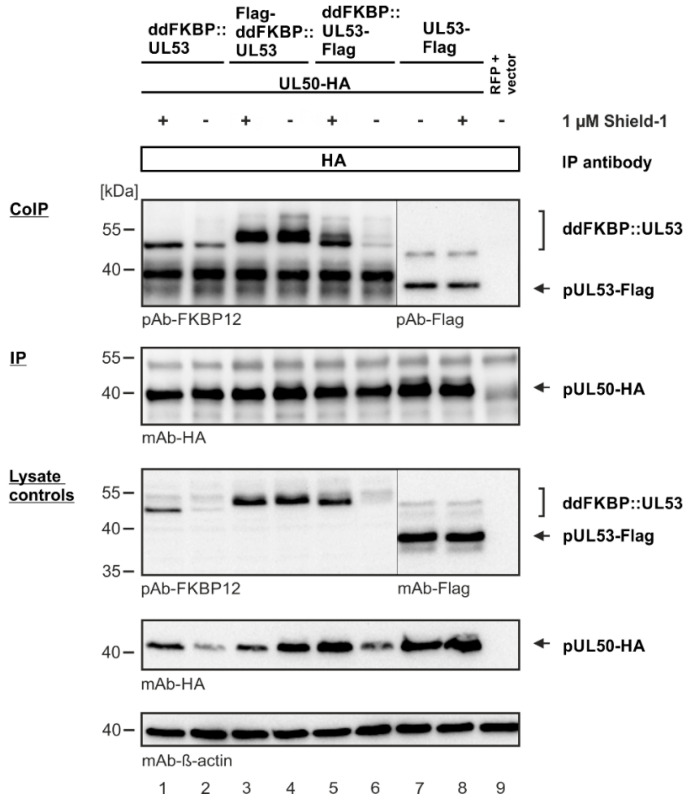
Interaction of ddFKBP::UL53 fusion proteins with pUL50-HA. 293T cells were transiently transfected with either the constructs coding for the three different ddFKBP::UL53 versions alone or in combination with pUL50-HA. Coexpression of the original core NEC heterodimer, pUL50-HA + pUL53-Flag, was used as a positive control; RFP + vector (pcDNA3.1) served as a negative control. To investigate protein stability, each fusion protein was expressed in the presence or in the absence of Shield-1 (1 µM). At 2 d p.t., cells were lysed, lysate controls were taken, and pUL50-HA was immunoprecipitated using mAb-HA. CoIP samples, intended to demonstrate NEC-specific protein interaction, were subjected to standard Wb analysis using specific antibodies as indicated.

The coding sequence of ddFKBP::UL53 was additionally cloned into a transient expression vector to analyze the mode and reliability of conditional expression in the absence of viral replication. For this purpose, three different fusion constructs harboring an N- or C-terminal Flag-tag were generated (Figure 12C). The expression and interaction properties of these constructs were used for cotransfection together with UL50-HA analyzed in 293T cells. To investigate the control of protein stability, each fusion protein was expressed both in the presence and absence of 1 µM of Shield-1. At 2 d p.t., total lysates were prepared from these cells, and CoIP was performed and analyzed by Western blot (Wb) staining. Cytotoxic effects of Shield-1 on four different relevant cell types were determined by a Neutral Red assay to exclude system-inherent artifacts, and data confirmed a low cytotoxic potential (Figure S6). Due to the fusion with ddFKBP, the ddFKBP::UL53 fusion proteins showed slower-migrating Wb bands than pUL53-Flag (Figure 13). All three fusion proteins were expressed but displayed different sensitivities to Shield-1 stabilization (Figure 13, lanes 1–6), in contrast to the Shield-1-unaffected expression of pUL53-Flag (Figure 13, lanes 7–8). As a consequence of the low level of destabilized pUL53 fusion proteins (lanes 2 and 6), also, the signal strength of pUL50 was reduced, since pUL50 and pUL53 can additionally stabilize each other upon heterodimerization [44,66]. In comparison to the unmodified proteins (lanes 7–8), the differentially stabilized ddFKBP::UL53 fusion proteins also led to variable CoIP signals of interaction with pUL50-HA. In the absence of Shield-1, the products ddFKBP::UL53 (lanes 1–2) and ddFKBP::UL53-Flag (lanes 5–6) were destabilized, while the N-terminally tagged version remained unaffected (lanes 3–4).

In order to ensure the proper functionality of the constructs used, the intranuclear colocalization properties between the ddFKBP::UL53 fusion proteins and pUL50-HA were examined (Figure S7). This was based on the known recruitment of pUL53 to the membrane-anchored pUL50, in the form of a characteristic NEC nuclear rim formation [11,19,38,44]. For this purpose, the respective constructs were cotransfected into HeLa cells, optionally under 1 µM of Shield-1 induction, to be used for NEC-specific immunofluorescence staining and subsequent confocal imaging. By addition of Shield-1, the ddFKBP::UL53 fusion proteins were detectable (Figure S7, images 1, 17 and 33), while a very weak or no signal was obtained in the absence of Shield-1 (images 5, 21 and 37). Importantly, by coexpression with pUL50-HA, the stabilized ddFKBP::UL53 fusion proteins were strictly recruited towards a nuclear rim colocalization between pUL50 and pUL53 (images 11, 27 and 43). Summarized, these results verify the reliability of the conditional expression, nuclear localization and interaction properties of the ddFKBP::UL53 fusion proteins.

**Figure 14:**
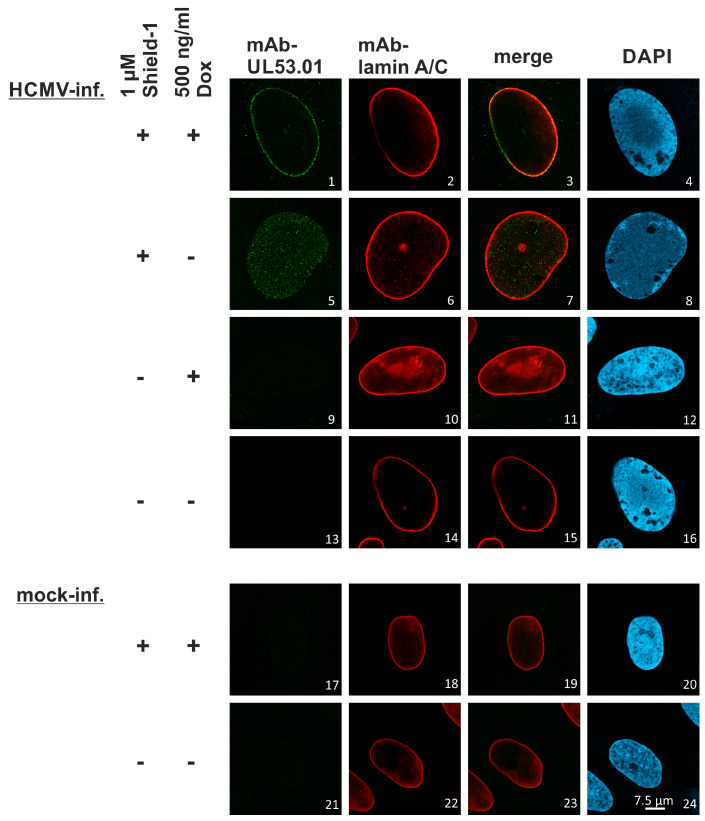
Shield-1- and Dox-controlled expression of viral core NEC proteins during HCMV ∆UL50-ΣUL53 infection. HFF-UL50 cells were infected with HCMV ∆UL50-ΣUL53 at MOI of 0.3. To analyze the experimental controllability of viral core NEC expression and intranuclear localization, 1 µM of Shield-1 and/or 500 ng/mL of Dox (refreshed every second day) were added as indicated. Uninfected cells served as a negative control (mock). At 6 d p.i., cells were fixed, used for an immunofluorescence staining by the indicated antibodies and analyzed by confocal imaging. Lamin A/C-specific counterstaining was used as a marker of the nuclear rim, representing the typical localization site of viral pUL50–pUL53 recruitment; DAPI counterstaining was used to monitor the morphologies of cell nuclei. For raw data, see https://doi.org/10.5281/zenodo.7794233 accessed on 3 April 2023.

The reconstituted stock virus of HCMV ΔUL50-ΣUL53 was then analyzed under specified conditions to ensure the aspired controllability of ddFKBP::UL53 during virus infection. For this purpose, HFF-UL50 cells were infected with HCMV ΔUL50-ΣUL53 at MOI 0.3 and were maintained under varied conditions using 1 µM Shield-1 (± Shield-1) and/or 500 ng/mL Dox (± Dox; refreshed every second day). At 6 d p.i., cells were fixed and used for NEC-specific immunofluorescence staining analyzed by confocal imaging (Figure 14). The staining of lamin A/C served as a marker for the typical nuclear rim morphology. In the presence of Shield-1, ddFKBP::UL53 was stabilized in HCMV-infected cells (images 1 and 5), whereas in the absence of Shield-1, no signal for pUL53 was detectable (images 9 and 13). In the pUL50-complementing setting (+Dox), the stabilized ddFKBP::UL53 was strictly recruited to the nuclear rim (image 3), whereas in the absence of Dox, the fusion protein was found diffusely distributed throughout the nucleus (image 7). Together, experimental infection with the recombinant HCMV ΔUL50-ΣUL53 indicated a reliable core NEC colocalization, including ddFKBP::UL53 under conditional expression regulated by Shield-1.

**Figure 15:**
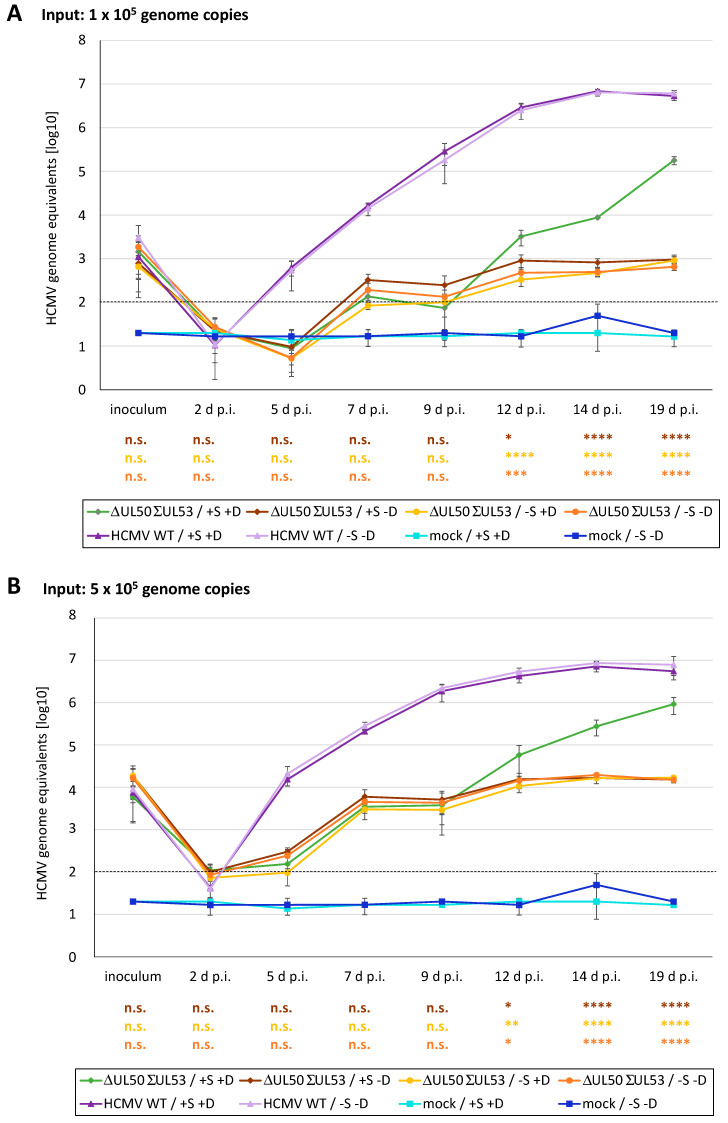
HCMV ∆UL50-ΣUL53 replication kinetics in HFF-UL50 cells applying different Shield-1 and Dox conditions. HFF-UL50 cells were cultivated in 24-well plates and infected with HCMV ∆UL50-ΣUL53 or parental HCMV AD169, using adjusted genome copy numbers, or remained uninfected (mock). Viral input was adjusted to 1 × 10^5^ (**A**) or 5 × 10^5^ genome equivalents (**B**). Infected cells were maintained under variable conditions, including the indicated combinations of Shield-1 (± Shield-1, 1 µM) and Dox (± Dox, 500 ng/mL) addition. At indicated time points, supernatants were harvested, and viral genome equivalents were determined using HCMV IE1-specific qPCR. Each condition was examined in biological triplicates, followed by qPCR measurements in additional technical duplicates; mean values SDs are shown. The standard containing 10^2^ HCMV DNA copies reached the cycle threshold at cycle 38 and was therefore defined as the limit of detection for qPCR, as shown by the black dashed line. Statistical analysis was performed using ordinary one-way ANOVA and post hoc Tukey testing on ∆UL50-ΣUL53 (+S −D, −S +D and −S −D) values in relation to ∆UL50-ΣUL53 +S +D; * *p* ≤ 0.05, ** *p* ≤ 0.01, *** *p* ≤ 0.001, **** *p* ≤ 0.0001; n.s., not significant.

In the next step, the replication efficiency of the HCMV ΔUL50-ΣUL53 under the various conditions was analyzed in comparison to the parental HCMV AD169 (WT) (Figure 15). To this end, HFF-UL50 cells were infected with HCMV ΔUL50-ΣUL53 or WT, using viral stocks adjusted to identical genome levels. In two parallel approaches, viral inocula representing 1 × 10^5^ (Figure 15A) or 5 × 10^5^ (Figure 15B) genomic copies of the respective virus stock were used for infection, as performed under the four different conditions to complement pUL50 (± 500 ng/mL of Dox) and to stabilize pUL53 (± 1 µM of Shield-1). Each condition was analyzed in triplicates, and supernatants were harvested at the indicated time points for HCMV genome-specific qPCR, as performed in additional technical duplicates. In parallel, a Neutral Red assay was performed to rule out possible cytotoxic effects of Shield-1 (Figure S6). In general, the input of 5 × 10^5^ genomes (Figure 15B) led to approx. one log level higher numbers of HCMV ΔUL50ΣUL53 genome equivalents at peak levels compared to 1 × 10^5^ (Figure 15A) genomes. The parental WT virus, instead, did not show Shield-1/Dox dependence and reached an early maximum of approx. 10^7^ genome equivalents. HCMV ΔUL50-ΣUL53 could be functionally complemented through +Dox pUL50 and +Shield-1-stabilized ddFKBP::UL53 (green curves), but nevertheless showed substantially lower replication levels than WT (Figure 15A,B; light purple and dark purple curves; statistical significance versus WT was *p* ≤ 0.0001, not shown). Until 9 d p.i., the varied conditions of Shield-1 and Dox did not lead to relevant differences in HCMV ΔUL50-ΣUL53 replication. However, from 12 to 19 d p.i., a significant increase in viral genome equivalents was noted for conditions of optimal rescue (Figure 15A,B; +S +D, green curves) compared to those conditions under which only one or none of the NEC proteins was expressed (Figure 15A,B; yellow, orange or brown curves, respectively). At 19 d p.i., the settings with no or partial Shield-1/Dox-mediated rescue remained approximately two log stages below optimal rescue. Summarized, these results indicate that both NEC proteins, pUL50 and pUL53, represent rate-limiting determinants of viral replication efficiency, i.e., their conditional down-modulation led to partial replication deficiency. Next, this recombinant virus was applied for the characterization of core NEC-inhibitory small molecules.

### 3.7. Utilization of the Conditionally NEC-Expressing HCMV ΔUL50-ΣUL53 for Experimental Confirmation of the Core NEC-Directed Inhibitory Activity of Ibrutinib

**Figure 16:**
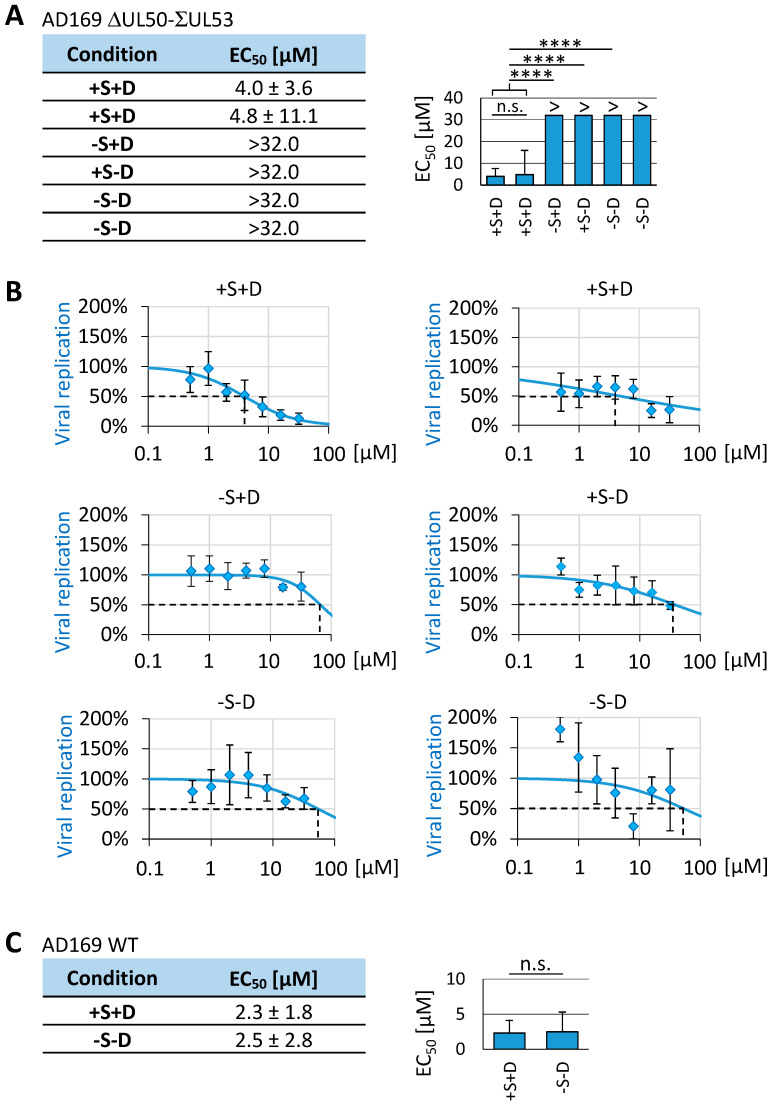
Loss of antiviral efficacy of ibrutinib in HCMV ∆UL50-ΣUL53-infected cells under conditions that did not allow the expression of viral core NEC proteins as a drug target. HFF-UL50 cells were cultivated in 96-well plates and infected with (**A**,**B**) HCMV ∆UL50-ΣUL53, with (**C**) parental HCMV AD169 (WT), using adjusted genome copy numbers, or remained uninfected (mock). Viral input was adjusted to 1 × 10^5^ copies per well. Infected cells were treated with a serial concentration of ibrutinib ranging from 0.5 µM to 50.0 µM. Infected and treated cells were maintained under variable conditions, including the indicated combinations of Shield-1 (±Shield-1, 1 µM) and Dox (±Dox, 500 ng/mL) addition. Dox was refreshed every second day. At 14 d p.i., supernatants were harvested, and viral genome equivalents were determined using HCMV IE1-specific qPCR. Each condition was examined in biological quadruplicates; mean values ± SDs are shown. In addition, the conditions, +S + D and −S −D, were given by two independent experimental replicates (whereby the second −S −D setting (panel B, lower right) had the highest SD and was excluded from statistics shown in panel (**A**)). Statistical analysis was performed using ordinary one-way ANOVA and post hoc Tukey testing; **** *p* ≤ 0.0001; n.s., not significant.

Finally, we aimed at a confirmation of the NEC-specific antiviral mechanism of the warhead ibrutinib. To this end, the conditional expression system of the HCMV ΔUL50-ΣUL53 recombinant was applied. The rationale was based on the concept that, suggesting an NEC-directed antiviral mode-of-action (MoA) of this drug, its activity should be markedly decreased under conditions lacking the expression of viral NEC proteins as targets. Under these conditions (without Dox/−D, i.e., lacking pUL50 induction, or without Shield-1/-S, i.e., lacking pUL53 stabilization), the postulated targets should be missing, thus preventing an NEC-specific antiviral MoA of ibrutinib. As depicted by the viral replication kinetics in Figure 15, replication of the HCMV recombinant ΔUL50-ΣUL53 under conditions of NEC deficiency (−S −D, +S −D or −S +D), was restricted to a low-level residual efficiency. On this basis, we specifically assessed the ibrutinib activity under such conditions of NEC-depleted, residual HCMV replication. The relative differences in viral replication efficiencies, under these four chosen conditions, were normalized by two means, namely, by using the same conditions in the DMSO control panel (±S, ±D) and by evaluating viral replication in percentage values (% DMSO). As an important finding, the lack of either one (+S −D or −S +D) or both of the viral core NEC proteins (−S −D) produced a statistically significant rise in the ibrutinib EC_50_ level (>32.0 µM, i.e., ranging above the highest analyzed concentration; Figure 16A,B). This was compared to the normal, NEC-positive reference conditions (+S + D) resulting in EC_50_ values of 4.0 ± 3.6 µM and 4.8 ± 11.1 µM in two biological replicates of this qPCR-based setting (Figure 16A,B). The parental wildtype, HCMV AD169, did not significantly respond to +S+D conditions in its ibrutinib sensitivity as expected (Figure 16C). Interestingly, HCMV recombinant ∆UL50-ΣUL53 showed a mean 1.83-fold increase in the EC_50_ value of ibrutinib (mean: 4.4 µM) over parental HCMV AD169 (mean: 2.4 µM) under +S + D conditions, which is possibly explained by the experimental pUL50 overexpression. These data provided a confirmation that the antiviral MoA of ibrutinib is, at least in part, target-specified towards one or both of the viral core NEC proteins.

## 4. Conclusions

The nuclear egress of viral capsids is a rate-limiting step during the lytic replication and production of infectious progeny of all herpesviruses [11,32]. The core NEC components and regulators are represented by the HCMV pUL50 and pUL53 proteins or the respective herpesviral homologs [11,16,32]. Recent achievements of our group and other researchers have illustrated the functional relevance of herpesviral core NECs, together with the associated proteins of the higher-order multicomponent NECs. Consequently, these proteins are considered as upcoming antiviral targets for next-generation antiherpesviral drugs [18,21,26,34,40,46]. Here, we focused on the use of covalently binding warheads and the discussion about their usefulness in the generation of NEC-directed antiviral small molecules.

In general, covalent protein ligands were avoided for a long period of time due to potential toxicity issues related to their limited specificity. The advance of the field offered a deeper understanding of the binding mechanism, leading to effective design principles. As a result, it became easier to design target-specific covalent inhibitors, thereby minimizing the chance of unwanted side effects. The majority of warheads are reported to react with a single target residue; however, this does not mean that warheads with promiscuous reactivity should be excluded from drug development projects. Current developments in target specificity, together with the advantages of irreversible inhibition, have made covalent inhibitors more and more scientifically attractive in the last decade [29,47,48,58]. In addition, the repertoire of drugs that has been developed against various human diseases is immense [78]. The modern approaches include both classical mechanistic properties of drugs and novel targeting strategies, including covalently binding drugs [47,79,80,81].

Broadening the toolbox of warheads that exert antiviral properties could result in novel inhibitors even for challenging targets. It is a highly interesting aspect that several of these warhead drugs, such as afatinib, ibrutinib and others, which have primarily been developed as covalent inhibitors of tumor-associated kinases and other clinically relevant targets [29], possess a marked antiviral potency. The data of this study provide evidence that warheads can be directed even to non-kinase targets and may particularly exert antiviral activity based on the inhibition of PPI. Here, PPI-targeted inhibition has been demonstrated in terms of a block in HCMV core NEC formation, which has recently been characterized as a rate-limiting step in viral replication efficiency [32,38,45]. Current experimental points of evidence that argue for the anti-HCMV activity, and, in particular, for the NEC-directed mode of activity of ibrutinib and other analyzed warheads include: (i) their strong efficacy in antiviral assays at non-cytotoxic concentrations, (ii) drug-mediated disruption of the typical NEC rim formation, (iii) inhibition of NEC interaction signals measured in PPI evaluation assays, and (iv) loss of antiviral activity against a recombinant HCMV with conditionally down-modulated NEC expression. Notably, our structural and bioinformatic analyses suggested potential target cysteine residues of the viral core NEC proteins that may serve as acceptors of warhead attack. First, Cys54 in pUL50 represents a structure-determining residue in the NEC groove formation and is thus primarily considered as a warhead-targeted candidate site. Secondly, although positioned downstream of the main hook element of pUL53 (defined by amino acids 55–87; [36]), three strictly conserved residues of Cys106, Csy122 and Cys125 have been considered as part of a structurally important zinc-binding site (zinc finger; [43]), and may likewise serve as warhead acceptor functionalities [11,16,41,42].

In the present study, the antiviral in vitro efficacy of warheads ranged, with slight variations for strains of HCMV, between 0.3 ± 0.1 µM (neratinib), 1.0 ± 0.2 µM (afatinib) and 16.0 ± 6.0 µM (ibrutinib) in GFP/YFP reporter assays (Figure 7), with a mean of 2.4 ± 2.3 µM (ibrutinib) in qPCR-based assays (Figure 16). Interestingly, the EC_50_ value of ibrutinib showed a mean 1.83-fold increase with the HCMV recombinant ∆UL50-ΣUL53 under +S + D conditions compared to the parental wildtype HCMV AD169, which might reflect increased warhead consumption under conditions of target pUL50/+D overexpression. Most importantly, the lack of either one (+S −D, −S + D) or both of the viral core NEC proteins (−S −D) produced a statistically significant rise in the ibrutinib EC_50_ level. It should be emphasized that previous studies described that various defects in regular core NEC functionality, in the case of HCMV or other herpesviruses, can lead to a viral switch to an NEC-independent, alternative mode of nucleocytoplasmic capsid release. This mode, however, appeared to be poorly regulated and rather based on cell lysis with massive nuclear envelope breakdown (NEBD; [32,38,82,83]), such that viral replication remained limited to a low-level residual efficiency. Thus, when assessing ibrutinib activity under these conditions of low-level, NEC-defective HCMV replication, the drug’s antiviral potency was found to be significantly reduced. This underlined the experimental postulate that ibrutinib has target specificity towards one or both of the viral core NEC proteins. In essence, our data highlight that selected warheads show strong anti-HCMV activity on the basis of core NEC-blocking properties, and the mechanistic features of the antiviral potential were illustrated by the use of a conditionally NEC-expressing, recombinant HCMV. In conclusion, these novel results underline the important, rate-limiting function of the HCMV core NEC as a drug-accessible determinant of viral replication. Future studies may add to the suggested strategy to exploit such NEC-directed warheads as potential candidates for innovative antiherpesviral drug targeting.

## Data Availability

The responsible authors declare that this article fully complies with the Data Availability Statements in the “MDPI Research Data Policies” section at https://www.mdpi.com/ethics (accessed on 4 April 2023).

## Supplementary Materials

The following are available online at https://www.mdpi.com/article/10.3390/cells12081162/s1, Table S1. Oligonucleotide primers used in this study, Figure S1: Impact of warhead compounds possessing antiviral activity on the localization of HCMV pUL53 (grayscale images), Figure S2: Assessment of the levels of cytotoxicity of clinically relevant warheads, Figure S3: Coimmunoprecipitation analysis: inhibitory impact of ibrutinib on core NEC interaction, Figure S4: Inhibitory impact of hit compound ibrutinib on the localization of three different herpesviral core NECs in plasmid-cotransfected cells (grayscale images), Figure S5: Inhibitory impact of hit compound ibrutinib on the localization of HCMV pUL53 (grayscale images), Figure S6: Analysis of putative cytotoxicity of the synthetic ligand Shield-1 on different cell types, Figure S7: Colocalization of transiently expressed FKBP::UL53 constructs with the core NEC interaction partner pUL50.

## References

[1] Goodrum F., Britt W., Mocarski E.S. (2021). Cytomegalovirus. Fields Virology.

[2] Boehmer P.E., Nimonkar A.V. (2003). Herpes virus replication. IUBMB Life.

[3] Whitley R.J. (1996). Herpesviruses. Medical Microbiology.

[4] Gugliesi F., Coscia A., Griffante G., Galitska G., Pasquero S., Albano C., Biolatti M. (2020). Where do we Stand after Decades of Studying Human Cytomegalovirus?. Microorganisms.

[5] Revello M.G., Gerna G. (2002). Diagnosis and management of human cytomegalovirus infection in the mother, fetus, and newborn infant. Clin. Microbiol. Rev..

[6] Tsutsui Y. (2009). Effects of cytomegalovirus infection on embryogenesis and brain development. Congenit. Anom..

[7] Avery R.K., Alain S., Alexander B.D., Blumberg E.A., Chemaly R.F., Cordonnier C., Duarte R.F., Florescu D.F., Kamar N., Kumar D. (2022). Maribavir for Refractory Cytomegalovirus Infections With or Without Resistance Post-Transplant: Results From a Phase 3 Randomized Clinical Trial. Clin. Infect. Dis..

[8] Hamilton S.T., Marschall M., Rawlinson W.D. (2020). Investigational Antiviral Therapy Models for the Prevention and Treatment of Congenital Cytomegalovirus Infection during Pregnancy. Antimicrob. Agents Chemother..

[9] Perera M.R., Wills M.R., Sinclair J.H. (2021). HCMV Antivirals and Strategies to Target the Latent Reservoir. Viruses.

[10] Pante N., Kann M. (2002). Nuclear pore complex is able to transport macromolecules with diameters of ~39 nm. Mol. Biol. Cell.

[11] Marschall M., Häge S., Conrad M., Alkhashrom S., Kicuntod J., Schweininger J., Kriegel M., Losing J., Tillmanns J., Neipel F. (2020). Nuclear Egress Complexes of HCMV and Other Herpesviruses: Solving the Puzzle of Sequence Coevolution, Conserved Structures and Subfamily-Spanning Binding Properties. Viruses.

[12] Draganova E.B., Valentin J., Heldwein E.E. (2021). The Ins and Outs of Herpesviral Capsids: Divergent Structures and Assembly Mechanisms across the Three Subfamilies. Viruses.

[13] Krug L.T., Pellett P.E. (2021). The Family Herpesviridae: A Brief Introduction. Fields Virology.

[14] Bigalke J.M., Heldwein E.E. (2017). Have NEC Coat, Will Travel: Structural Basis of Membrane Budding During Nuclear Egress in Herpesviruses. Adv. Virus Res..

[15] Lye M.F., Wilkie A.R., Filman D.J., Hogle J.M., Coen D.M. (2017). Getting to and through the inner nuclear membrane during herpesvirus nuclear egress. Curr. Opin. Cell Biol..

[16] Marschall M., Muller Y.A., Diewald B., Sticht H., Milbradt J. (2017). The human cytomegalovirus nuclear egress complex unites multiple functions: Recruitment of effectors, nuclear envelope rearrangement, and docking to nuclear capsids. Rev. Med. Virol..

[17] Roller R.J., Baines J.D. (2017). Herpesvirus Nuclear Egress. Adv. Anat. Embryol. Cell Biol..

[18] Marschall M., Marzi A., aus dem Siepen P., Jochmann R., Kalmer M., Auerochs S., Lischka P., Leis M., Stamminger T. (2005). Cellular p32 recruits cytomegalovirus kinase pUL97 to redistribute the nuclear lamina. J. Biol. Chem..

[19] Milbradt J., Auerochs S., Marschall M. (2007). Cytomegaloviral proteins pUL50 and pUL53 are associated with the nuclear lamina and interact with cellular protein kinase C. J. Gen. Virol..

[20] Milbradt J., Auerochs S., Sticht H., Marschall M. (2009). Cytomegaloviral proteins that associate with the nuclear lamina: Components of a postulated nuclear egress complex. J. Gen. Virol..

[21] Milbradt J., Kraut A., Hutterer C., Sonntag E., Schmeiser C., Ferro M., Wagner S., Lenac T., Claus C., Pinkert S. (2014). Proteomic analysis of the multimeric nuclear egress complex of human cytomegalovirus. Mol. Cell Proteom..

[22] Milbradt J., Sonntag E., Wagner S., Strojan H., Wangen C., Rovis T.L., Lisnic B., Jonjic S., Sticht H., Britt W.J. (2018). Human Cytomegalovirus Nuclear Capsids Associate with the Core Nuclear Egress Complex and the Viral Protein Kinase pUL97. Viruses.

[23] Milbradt J., Webel R., Auerochs S., Sticht H., Marschall M. (2010). Novel mode of phosphorylation-triggered reorganization of the nuclear lamina during nuclear egress of human cytomegalovirus. J. Biol. Chem..

[24] Kicuntod J., Häge S., Hahn F., Sticht H., Marschall M. (2022). The Oligomeric Assemblies of Cytomegalovirus Core Nuclear Egress Proteins Are Associated with Host Kinases and Show Sensitivity to Antiviral Kinase Inhibitors. Viruses.

[25] Sonntag E., Milbradt J., Svrlanska A., Strojan H., Hage S., Kraut A., Hesse A.M., Amin B., Sonnewald U., Coute Y. (2017). Protein kinases responsible for the phosphorylation of the nuclear egress core complex of human cytomegalovirus. J. Gen. Virol..

[26] Milbradt J., Hutterer C., Bahsi H., Wagner S., Sonntag E., Horn A.H., Kaufer B.B., Mori Y., Sticht H., Fossen T. (2016). The Prolyl Isomerase Pin1 Promotes the Herpesvirus-Induced Phosphorylation-Dependent Disassembly of the Nuclear Lamina Required for Nucleocytoplasmic Egress. PLoS Pathog..

[27] Steingruber M., Marschall M. (2020). The Cytomegalovirus Protein Kinase pUL97:Host Interactions, Regulatory Mechanisms and Antiviral Drug Targeting. Microorganisms.

[28] Wild M., Hahn F., Bruckner N., Schutz M., Wangen C., Wagner S., Sommerer M., Strobl S., Marschall M. (2022). Cyclin-Dependent Kinases (CDKs) and the Human Cytomegalovirus-Encoded CDK Ortholog pUL97 Represent Highly Attractive Targets for Synergistic Drug Combinations. Int. J. Mol. Sci..

[29] Peczka N., Orgovan Z., Abranyi-Balogh P., Keseru G.M. (2022). Electrophilic warheads in covalent drug discovery: An overview. Expert Opin. Drug Discov..

[30] Petri L., Egyed A., Bajusz D., Imre T., Hetenyi A., Martinek T., Abranyi-Balogh P., Keseru G.M. (2020). An electrophilic warhead library for mapping the reactivity and accessibility of tractable cysteines in protein kinases. Eur. J. Med. Chem..

[31] Roskoski R. (2021). Orally effective FDA-approved protein kinase targeted covalent inhibitors (TCIs). Pharmacol. Res..

[32] Hage S., Marschall M. (2022). ‘Come together’—The Regulatory Interaction of Herpesviral Nuclear Egress Proteins Comprises Both Essential and Accessory Functions. Cells.

[33] Marschall M., Stamminger T. (2009). Molecular targets for antiviral therapy of cytomegalovirus infections. Future Microbiol..

[34] Alkhashrom S., Kicuntod J., Stillger K., Lutzenburg T., Anzenhofer C., Neundorf I., Marschall M., Eichler J. (2022). A Peptide Inhibitor of the Human Cytomegalovirus Core Nuclear Egress Complex. Pharmaceuticals.

[35] Kicuntod J., Hage S., Losing J., Kopar S., Muller Y.A., Marschall M. (2023). An antiviral targeting strategy based on the inducible interference with cytomegalovirus nuclear egress complex. Antivir. Res..

[36] Lösing J., Häge S., Schütz M., Wagner S., Wardin J., Sticht H., Marschall M. (2022). ‘Shared-Hook’ and ‘Changed-Hook’ Binding Activities of Herpesviral Core Nuclear Egress Complexes Identified by Random Mutagenesis. Cells.

[37] Schütz M., Müller R., Socher E., Wangen C., Full F., Wyler E., Wong D., Scherer M., Stamminger T., Chou S. (2022). Highly Conserved Interaction Profiles between Clinically Relevant Mutants of the Cytomegalovirus CDK-like Kinase pUL97 and Human Cyclins: Functional Significance of Cyclin H. Int. J. Mol. Sci..

[38] Häge S., Buscher N., Pakulska V., Hahn F., Adrait A., Krauter S., Borst E.M., Schlotzer-Schrehardt U., Coute Y., Plachter B. (2021). The Complex Regulatory Role of Cytomegalovirus Nuclear Egress Protein pUL50 in the Production of Infectious Virus. Cells.

[39] Häge S., Horsch D., Stilp A.C., Kicuntod J., Muller R., Hamilton S.T., Egilmezer E., Rawlinson W.D., Stamminger T., Sonntag E. (2020). A quantitative nuclear egress assay to investigate the nucleocytoplasmic capsid release of human cytomegalovirus. J. Virol. Method..

[40] Kicuntod J., Alkhashrom S., Hage S., Diewald B., Muller R., Hahn F., Lischka P., Sticht H., Eichler J., Marschall M. (2021). Properties of Oligomeric Interaction of the Cytomegalovirus Core Nuclear Egress Complex (NEC) and Its Sensitivity to an NEC Inhibitory Small Molecule. Viruses.

[41] Muller Y.A., Häge S., Alkhashrom S., Hollriegl T., Weigert S., Dolles S., Hof K., Walzer S.A., Egerer-Sieber C., Conrad M. (2020). High-resolution crystal structures of two prototypical β- and γ-herpesviral nuclear egress complexes unravel the determinants of subfamily specificity. J. Biol. Chem..

[42] Schweininger J., Kriegel M., Häge S., Conrad M., Alkhashrom S., Lösing J., Weiler S., Tillmanns J., Egerer-Sieber C., Decker A. (2022). The crystal structure of the varicella-zoster Orf24-Orf27 nuclear egress complex spotlights multiple determinants of herpesvirus subfamily specificity. J. Biol. Chem..

[43] Walzer S.A., Egerer-Sieber C., Sticht H., Sevvana M., Hohl K., Milbradt J., Muller Y.A., Marschall M. (2015). Crystal Structure of the Human Cytomegalovirus pUL50-pUL53 Core Nuclear Egress Complex Provides Insight into a Unique Assembly Scaffold for Virus-Host Protein Interactions. J. Biol. Chem..

[44] Häge S., Sonntag E., Borst E.M., Tannig P., Seyler L., Bauerle T., Bailer S.M., Lee C.P., Muller R., Wangen C. (2020). Patterns of Autologous and Nonautologous Interactions Between Core Nuclear Egress Complex (NEC) Proteins of α-, β- and γ-Herpesviruses. Viruses.

[45] Häge S., Sonntag E., Svrlanska A., Borst E.M., Stilp A.C., Horsch D., Muller R., Kropff B., Milbradt J., Stamminger T. (2021). Phenotypical Characterization of the Nuclear Egress of Recombinant Cytomegaloviruses Reveals Defective Replication upon ORF-UL50 Deletion but Not pUL50 Phosphosite Mutation. Viruses.

[46] Alkhashrom S., Kicuntod J., Häge S., Schweininger J., Muller Y.A., Lischka P., Marschall M., Eichler J. (2021). Exploring the Human Cytomegalovirus Core Nuclear Egress Complex as a Novel Antiviral Target: A New Type of Small Molecule Inhibitors. Viruses.

[47] Baillie T.A. (2016). Targeted Covalent Inhibitors for Drug Design. Angew. Chem. Int. Ed. Engl..

[48] Lonsdale R., Burgess J., Colclough N., Davies N.L., Lenz E.M., Orton A.L., Ward R.A. (2017). Expanding the Armory: Predicting and Tuning Covalent Warhead Reactivity. J. Chem. Inf. Model..

[49] Gehringer M., Laufer S.A. (2019). Emerging and Re-Emerging Warheads for Targeted Covalent Inhibitors: Applications in Medicinal Chemistry and Chemical Biology. J. Med. Chem..

[50] Zhang T., Hatcher J.M., Teng M., Gray N.S., Kostic M. (2019). Recent Advances in Selective and Irreversible Covalent Ligand Development and Validation. Cell Chem. Biol..

[51] Rowe W.P., Hartley J.W., Waterman S., Turner H.C., Huebner R.J. (1956). Cytopathogenic agent resembling human salivary gland virus recovered from tissue cultures of human adenoids. Proc. Soc. Exp. Boil. Med..

[52] Marschall M., Freitag M., Weiler S., Sorg G., Stamminger T. (2000). Recombinant green fluorescent protein-expressing human cytomegalovirus as a tool for screening antiviral agents. Antimicrob. Agents Chemother..

[53] Wagenknecht N., Reuter N., Scherer M., Reichel A., Muller R., Stamminger T. (2015). Contribution of the Major ND10 Proteins PML, hDaxx and Sp100 to the Regulation of Human Cytomegalovirus Latency and Lytic Replication in the Monocytic Cell Line THP-1. Viruses.

[54] Schregel V., Auerochs S., Jochmann R., Maurer K., Stamminger T., Marschall M. (2007). Mapping of a self-interaction domain of the cytomegalovirus protein kinase pUL97. J. Gen. Virol..

[55] Banaszynski L.A., Chen L.C., Maynard-Smith L.A., Ooi A.G., Wandless T.J. (2006). A rapid, reversible, and tunable method to regulate protein function in living cells using synthetic small molecules. Cell.

[56] Glaß M., Busche A., Wagner K., Messerle M., Borst E.M. (2009). Conditional and reversible disruption of essential herpesvirus proteins. Nat. Method..

[57] Tischer B.K., Smith G.A., Osterrieder N. (2010). En passant mutagenesis: A two step markerless red recombination system. Methods Mol. Biol..

[58] Johansson M.H. (2012). Reversible Michael additions: Covalent inhibitors and prodrugs. Mini-Rev. Med. Chem..

[59] Lorz K., Hofmann H., Berndt A., Tavalai N., Mueller R., Schlotzer-Schrehardt U., Stamminger T. (2006). Deletion of open reading frame UL26 from the human cytomegalovirus genome results in reduced viral growth, which involves impaired stability of viral particles. J. Virol..

[60] Repetto G., del Peso A., Zurita J.L. (2008). Neutral red uptake assay for the estimation of cell viability/cytotoxicity. Nat. Protoc..

[61] Sonntag E., Hamilton S.T., Bahsi H., Wagner S., Jonjic S., Rawlinson W.D., Marschall M., Milbradt J. (2016). Cytomegalovirus pUL50 is the multi-interacting determinant of the core nuclear egress complex (NEC) that recruits cellular accessory NEC components. J. Gen. Virol..

[62] Webel R., Milbradt J., Auerochs S., Schregel V., Held C., Nobauer K., Razzazi-Fazeli E., Jardin C., Wittenberg T., Sticht H. (2011). Two isoforms of the protein kinase pUL97 of human cytomegalovirus are differentially regulated in their nuclear translocation. J. Gen. Virol..

[63] Gerna G., Kabanova A., Lilleri D. (2019). Human Cytomegalovirus Cell Tropism and Host Cell Receptors. Vaccines.

[64] Sinzger C., Hahn G., Digel M., Katona R., Sampaio K.L., Messerle M., Hengel H., Koszinowski U., Brune W., Adler B. (2008). Cloning and sequencing of a highly productive, endotheliotropic virus strain derived from human cytomegalovirus TB40/E. J. Gen. Virol..

[65] Sievers F., Higgins D.G. (2014). Clustal omega. Curr. Protoc. Bioinform..

[66] Milbradt J., Auerochs S., Sevvana M., Muller Y.A., Sticht H., Marschall M. (2012). Specific residues of a conserved domain in the N terminus of the human cytomegalovirus pUL50 protein determine its intranuclear interaction with pUL53. J. Biol. Chem..

[67] Pettersen E.F., Goddard T.D., Huang C.C., Couch G.S., Greenblatt D.M., Meng E.C., Ferrin T.E. (2004). UCSF Chimera—A visualization system for exploratory research and analysis. J. Comput. Chem..

[68] Lanman B.A., Allen J.R., Allen J.G., Amegadzie A.K., Ashton K.S., Booker S.K., Chen J.J., Chen N., Frohn M.J., Goodman G. (2020). Discovery of a Covalent Inhibitor of KRAS(G12C) (AMG 510) for the Treatment of Solid Tumors. J. Med. Chem..

[69] Pellerino A., Soffietti R., Bruno F., Manna R., Muscolino E., Botta P., Palmiero R., Ruda R. (2022). Neratinib and Capecitabine for the Treatment of Leptomeningeal Metastases from HER2-Positive Breast Cancer: A Series in the Setting of a Compassionate Program. Cancers.

[70] Johnson A.R., Kohli P.B., Katewa A., Gogol E., Belmont L.D., Choy R., Penuel E., Burton L., Eigenbrot C., Yu C. (2016). Battling Btk Mutants with Noncovalent Inhibitors that Overcome Cys481 and Thr474 Mutations. ACS Chem. Biol..

[71] Cross D.A., Ashton S.E., Ghiorghiu S., Eberlein C., Nebhan C.A., Spitzler P.J., Orme J.P., Finlay M.R., Ward R.A., Mellor M.J. (2014). AZD9291, an irreversible EGFR TKI, overcomes T790M-mediated resistance to EGFR inhibitors in lung cancer. Cancer Discov..

[72] Liao X., Wu N., Liu D., Shuai B., Li S., Li K. (2020). Levodopa/carbidopa/entacapone for the treatment of early Parkinson’s disease: A meta-analysis. Neurol. Sci..

[73] Mathema V.B., Koh Y.S., Thakuri B.C., Sillanpaa M. (2012). Parthenolide, a sesquiterpene lactone, expresses multiple anti-cancer and anti-inflammatory activities. Inflammation.

[74] Fu Z., Li S., Liu J., Zhang C., Jian C., Wang L., Zhang Y., Shi C. (2022). Natural Product Alantolactone Targeting AKR1C1 Suppresses Cell Proliferation and Metastasis in Non-Small-Cell Lung Cancer. Front. Pharmacol..

[75] Wymann M.P., Bulgarelli-Leva G., Zvelebil M.J., Pirola L., Vanhaesebroeck B., Waterfield M.D., Panayotou G. (1996). Wortmannin inactivates phosphoinositide 3-kinase by covalent modification of Lys-802, a residue involved in the phosphate transfer reaction. Mol. Cell. Biol..

[76] Chien J.Y., Chou Y.Y., Ciou J.W., Liu F.Y., Huang S.P. (2021). The Effects of Two Nrf2 Activators, Bardoxolone Methyl and Omaveloxolone, on Retinal Ganglion Cell Survival during Ischemic Optic Neuropathy. Antioxidants.

[77] Schutz M., Steingruber M., Socher E., Muller R., Wagner S., Kogel M., Sticht H., Marschall M. (2021). Functional Relevance of the Interaction between Human Cyclins and the Cytomegalovirus-Encoded CDK-Like Protein Kinase pUL97. Viruses.

[78] Coen D.M., Richman D.D. (2013). Antiviral Agents. Fields Virology.

[79] Hagel M., Niu D., St Martin T., Sheets M.P., Qiao L., Bernard H., Karp R.M., Zhu Z., Labenski M.T., Chaturvedi P. (2011). Selective irreversible inhibition of a protease by targeting a noncatalytic cysteine. Nat. Chem. Biol..

[80] Ourique G.S., Vianna J.F., Neto J.X.L., Oliveira J.I.N., Mauriz P.W., Vasconcelos M.S., Caetano E.W.S., Freire V.N., Albuquerque E.L., Fulco U.L. (2016). A quantum chemistry investigation of a potential inhibitory drug against the dengue virus. Rsc. Adv..

[81] Zeng Q., Nair A.G., Rosenblum S.B., Huang H.C., Lesburg C.A., Jiang Y., Selyutin O., Chan T.Y., Bennett F., Chen K.X. (2013). Discovery of an irreversible HCV NS5B polymerase inhibitor. Bioorganic Med. Chem. Lett..

[82] Grimm K.S., Klupp B.G., Granzow H., Muller F.M., Fuchs W., Mettenleiter T.C. (2012). Analysis of viral and cellular factors influencing herpesvirus-induced nuclear envelope breakdown. J. Virol..

[83] Klupp B.G., Granzow H., Mettenleiter T.C. (2011). Nuclear envelope breakdown can substitute for primary envelopment-mediated nuclear egress of herpesviruses. J. Virol..

