# Supplementary material for: Assessment of Covalently Binding Warhead Compounds in the Validation of the Cytomegalovirus Nuclear Egress Complex as an Antiviral Target
Source: Cells. 2023 Apr 14;12(8):1162. doi: 10.3390/cells12081162 (PMC10137179; doi:10.3390/cells12081162)
Supplement: Supplementary file 1 [file cells-12-01162-s001.zip › cells-2212920-supplementary.pdf]

cells

Supplementary Materials

Assessment of covalently binding warhead compounds in the validation of the cytomegalovirus nuclear egress complex as an antiviral target

Julia Tillmanns [1], Sigrun Häge [1], Eva Maria Borst [2], Julia Wardin [1], Jan Eickhoff [3], Bert Klebl [3,4], Sabrina Wagner [1], Christina Wangen [1], Friedrich Hahn [1], Eileen Socher [5] and Manfred Marschall [1,*]

[1] Institute for Clinical and Molecular Virology, Friedrich-Alexander University of Erlangen-Nürnberg (FAU), Erlangen, Germany
[2] Institute of Virology, Hannover Medical School, Hannover, Germany
[3] Lead Discovery Center GmbH (LDC), Dortmund, Germany
[4] The Norwegian College of Fishery Science, UiT, The Arctic University of Norway, Tromsø, Norway
[5] Institute of Anatomy, Functional and Clinical Anatomy, FAU, Erlangen, Germany;

- Correspondence: manfred.marschall@fau.de; phone: +49-9131-8536096

Table S1. Oligonucleotide primers used in this study. The following information is given by the sequence description: coding sequences (capital letters), start and stop codons (capital letters underlined), restriction sites (capital letters bold), tag sequences (capital letters italic) and additional nucleotides (lower case letters).

Primer	Sequence (5′ → 3′)
**5-FKBP-UL53-EcoRI**	tac**GAATTC**ATGTCTAGCGTGGGAGTG
**3-UL53-XhoI**	tac**CTCGAG**TCAAGGCGCACGAATGCTG
**3-UL53-Flag-XhoI**	tac**CTCGAG**TCA*CTTGTCGTCATCGTCTTTGTAGTC*AGGCGCACGAATGCTGTTG
**5-Flag-FKBP-UL53-EcoRI**	tac**GAATTC**ATG*GACTACAAAGACGATGACGACAAG*TCTAGCGTGGGAGTGCAG
**5-PvuI cc EcoRI UL53**	gag**CGATCG**CC**GAATTC**ATGTCTAGCGTGAGC
**3-XbaI ApaI Flag**	gag**TCTAGAGGGCCC**TCA*CTTGTCGTCATC*
**5-CDK7-PvuI**	tgaC**GATCGC**CATGGCTCTGGACGTGAAGTCTC
**3-CDK7-SalI**	tga**GTCGAC**TTAAAAAATTAGTTTCTTGGGCAATCCTCCTTGTTCTAAGGC
**5-EcoRI-UL97 amino acid 231**	tga**GAATTC**CCGAAAGTCAGGACAGCGCCGT
**3-LgBit-UL97-Flag-1-280**	tga**GTCGACTCTAGA**TTA*CTTGTCGTCATCGTCTTTGTAGTC*GGGCCCTCTTGAGATGATCTGGTCGTTG
**5-Pvu-BamHI-CycH**	agt**CGATCG**CCGGATCCATGTACCACAACAGTAGTCAGAAGCGG
**3-SalI-XbaI-HA-CycH**	gta**GTCGACTCTAGA**TTA*AGCGTAATCTGGAACATCGTATGGG*TAGGGCCCTCTTGAGAGAGATTCTACCAGG

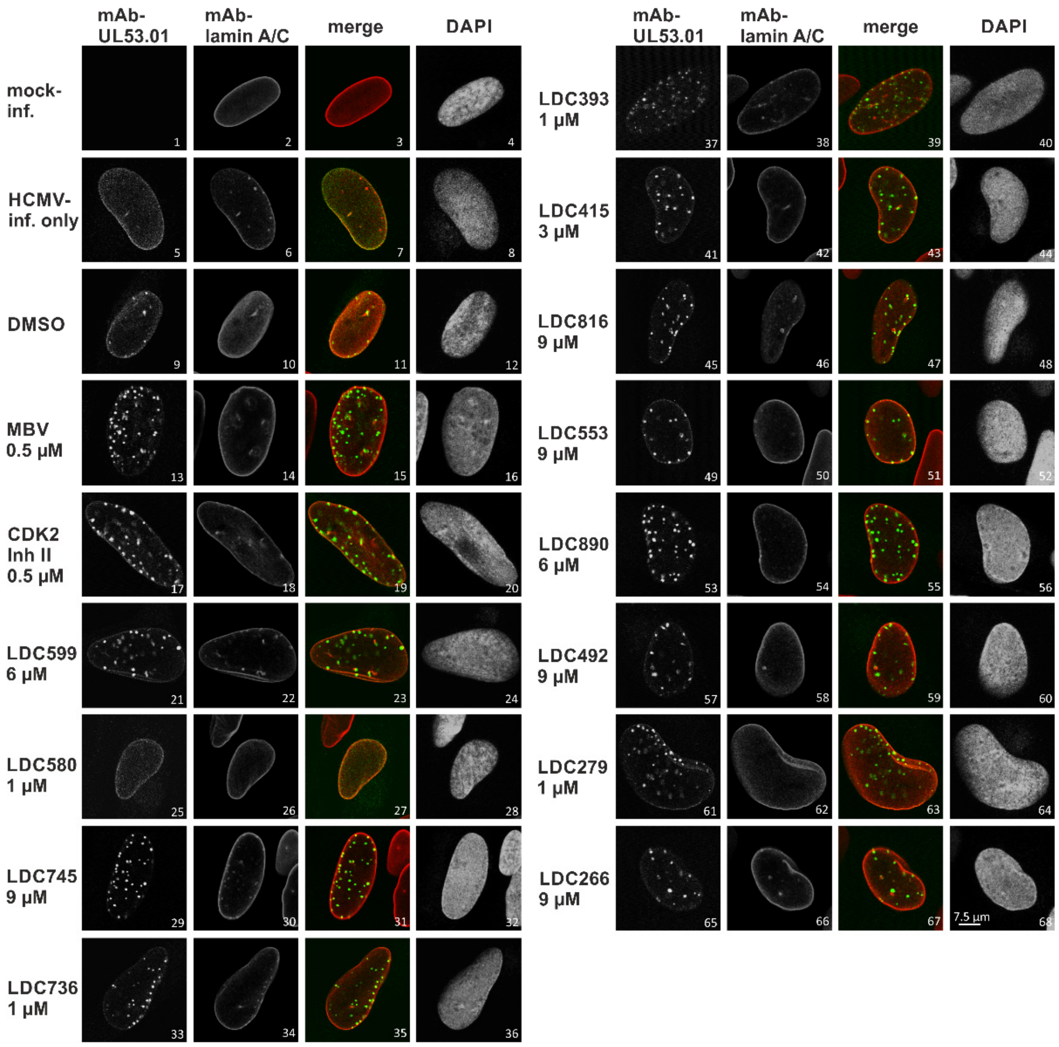

Figure S1. Impact of warhead compounds possessing antiviral activity on the localization of HCMV pUL53 (grayscale images). HFFs were infected with HCMV AD169 (MOI 0.1) and immediately treated with warhead compounds. Compounds were applied, as based on their individual EC_{50} values, at concentrations exerting partial-grade antiviral activity (see indicated), and the determination was performed in biological triplicates. At 5 d p.i., cells were fixed, used for IF staining with the indicated antibodies and analyzed for intranuclear localization by confocal imaging. DAPI counterstaining indicated the nuclei morphology of the respective cells. Additional single channel, grayscale images to Figure 4.

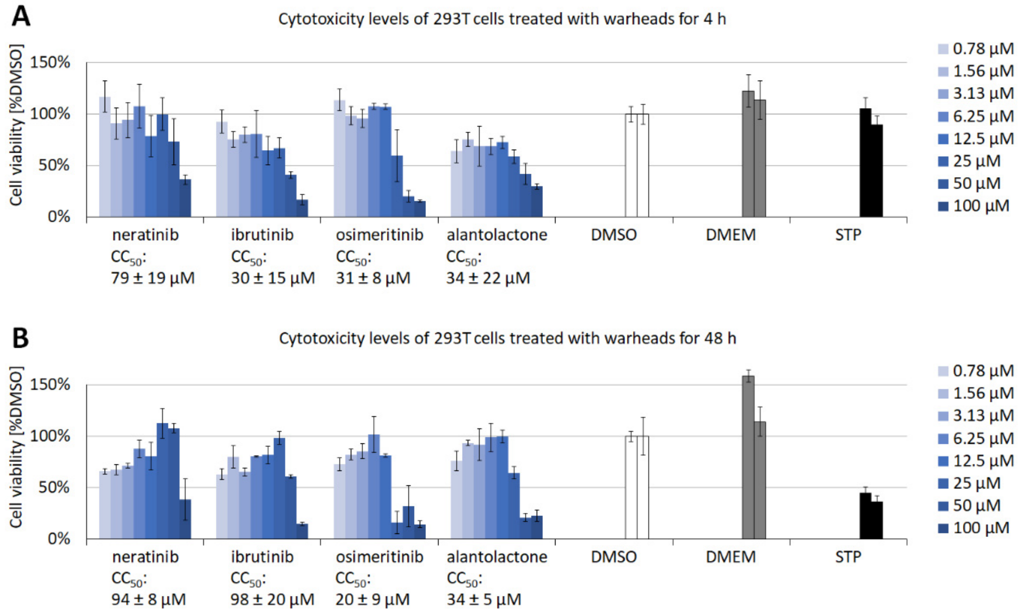

Figure S2. Assessment of the levels of cytotoxicity of clinically relevant warheads. To determine cytotoxicity levels of warhead compounds, 293T cells were treated with serial concentrations (0.78-100 µM as shown on the right), before cell viability was measured 4 h **(A)** or 48 h **(B)** post-treatment via Neutral Red staining. Staurosporine-treated cells (STP, 1 µM) cells served as a positive control, solvent-treated cells (DMSO) as a negative control.

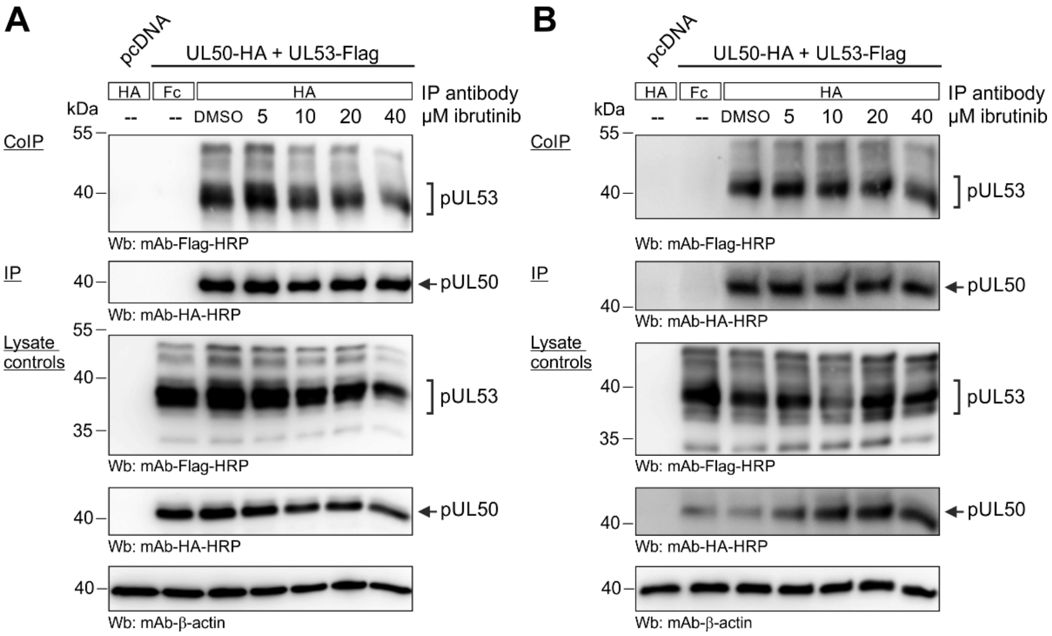

Figure S3. Coimmunoprecipitation analysis: inhibitory impact of ibrutinib on core NEC interaction. (A) Serial concentrations of ibrutinib were added to plasmid-cotransfected 293T cells (pcDNA-UL50-HA + pcDNA-UL53-Flag) at 24 h post-transfection (p.t.). **(B)** Alternatively, the identical ibrutinib concentrations were added after cell lysis and were maintained during the CoIP reaction. In both cases, total lysates were prepared at 48 h p.t., before CoIP analysis was performed under continued drug treatment. Lysate controls were taken, and pUL50-HA was immunoprecipitated using mAb-HA. Unspecific antibody binding was excluded using the mouse-derived Fc-fragment. CoIP samples, as intended to demonstrate NEC-specific interaction inhibition, were subjected to standard Wb analysis using specific antibodies as indicated.

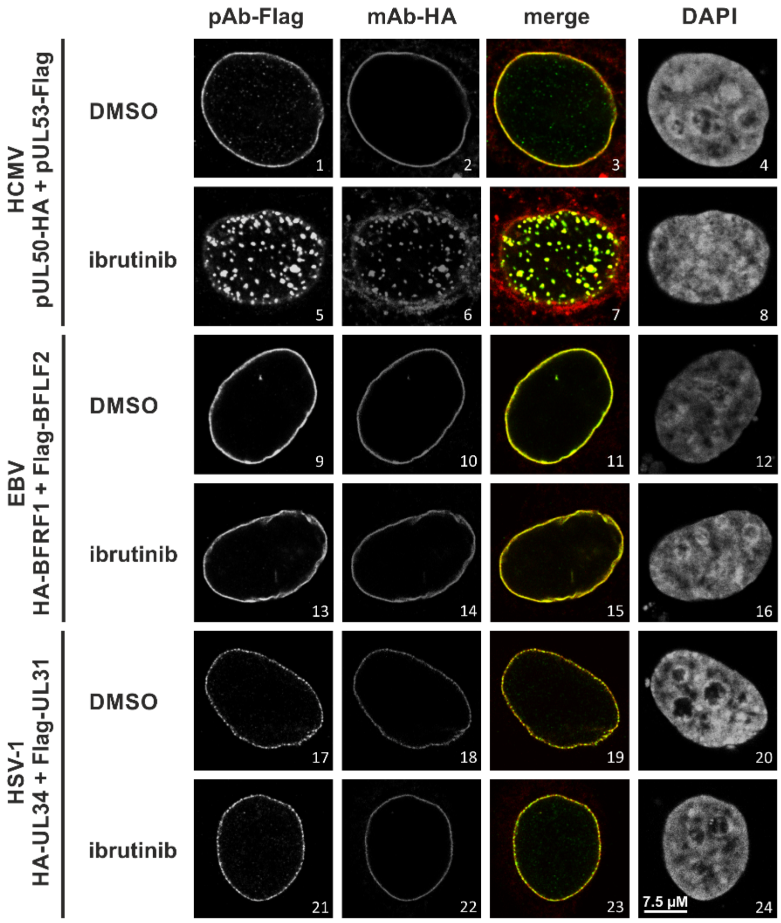

Figure S4. Inhibitory impact of hit compound ibrutinib on the localization of three different herpesviral core NECs in plasmid-cotransfected cells (grayscale images). HeLa cells were cotransfected with each two of the indicated NEC expression plasmids (HSV-1 HA-UL34 + Flag-UL31; HCMV pUL50-HA + pUL53-Flag, EBV HA-BFRF1 + Flag-BFLF2). Ibrutinib was applied at a concentration of 4 µM immediately after transfection. At 3 d post-transfection (p.t.), cells were fixed, used for IF staining with the indicated tag antibodies and analyzed for intranuclear localization by confocal imaging. DAPI counterstaining represented the nuclei morphology of the respective cells. Additional single channel, grayscale images to Figure 10.

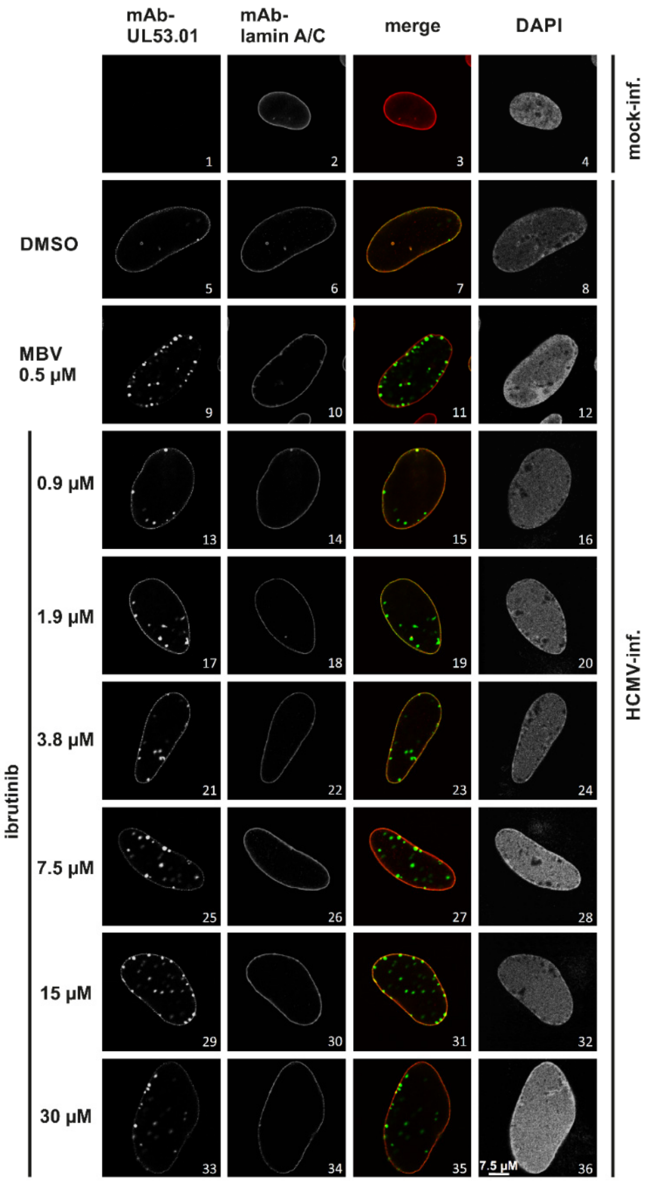

Figure S5. Inhibitory impact of hit compound ibrutinib on the localization of HCMV pUL53 (grayscale images). HFFs were infected with HCMV AD169 (MOI 0.1) and immediately treated with the warhead compound. Ibrutinib was applied at the indicated concentrations and the determination was performed in biological triplicates. At 5 d p.i., cells were fixed, used for IF staining with the indicated antibodies and analyzed for intranuclear localization by confocal imaging. DAPI counterstaining represented the nuclei morphology of the respective cells. Additional single channel, grayscale images to Figure 11.

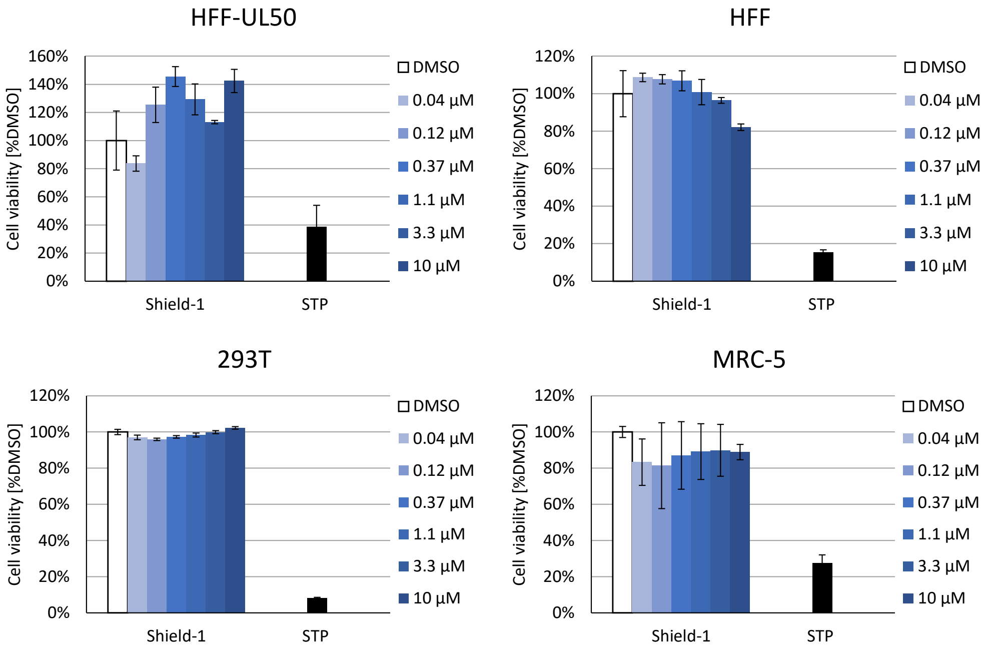

Figure S6. Analysis of putative cytotoxicity of the synthetic ligand Shield-1 on different cell types. Four different types of cells (recombinant complementing cells HFF-UL50, primary HFFs and and MRC-5 cells, and immortalized 293T) were treated with various concentrations of Shield-1 (0.04–10 µM as shown on the right). At 4 (293T) or 7 d post-treatment, cell viability was assessed by Neutral Red staining. Data are presented as mean values ± SD over three biological replicates. Cytotoxic staurosporine (STP, 1 µM) served as a positive control, DMSO-treated cells as a negative control.

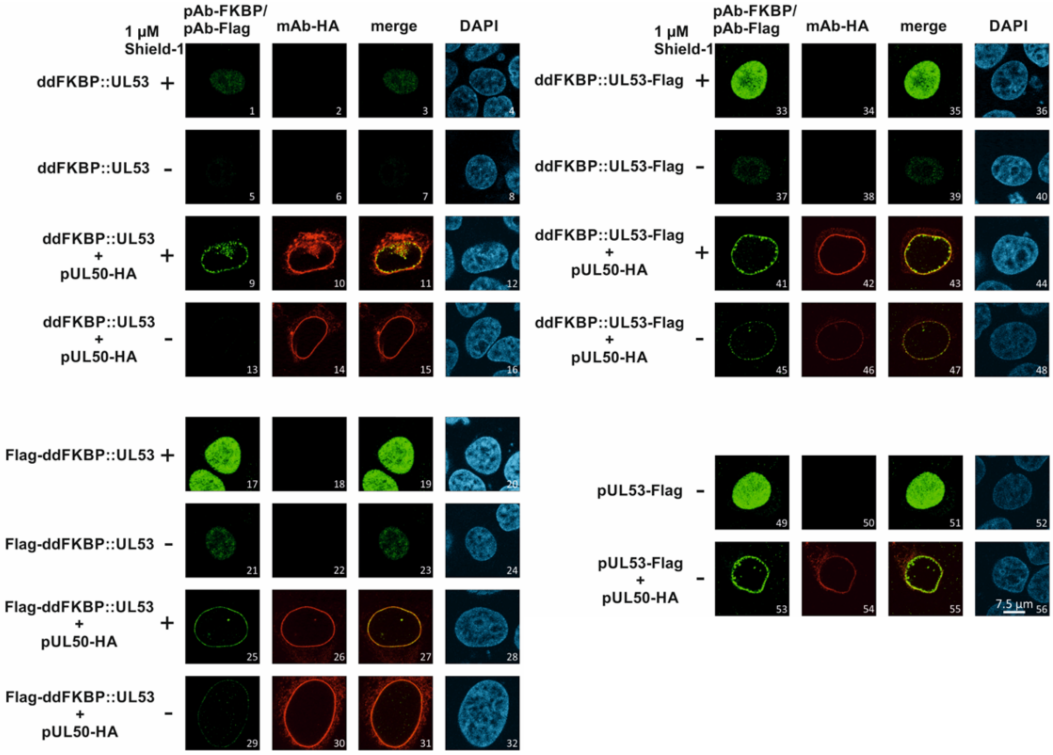

Figure S7. Colocalization of transiently expressed FKBP::UL53 constructs with the core NEC interaction partner pUL50. HeLa cells were seeded on coverslips, transiently transfected with one of the three different FKBP::UL53 expression constructs and in combination with the construct for pUL50-HA. Cotransfection of constructs expressing the parental pUL53-Flag and pUL50-HA served as a positive control for colocalization at the nuclear rim. The conditional way of expression and localization was investigated by the addition of 1 µM Shield-1. At 2 d p.i., cells were fixed, used for an immunostaining with tag- or protein-specific antibodies, as indicated on top of the panels, and analyzed by confocal imaging. DAPI counterstaining indicated the nuclei morphology of the respective cells.
